# Quantitative MS‐Based Proteomics: Comparing the MCF‐7 Cellular Response to Hypoxia and a 2‐Oxoglutarate Analogue

**DOI:** 10.1002/cbic.201900719

**Published:** 2020-03-03

**Authors:** Jacob T. Bush, Mun Chiang Chan, Shabaz Mohammed, Christopher J. Schofield

**Affiliations:** ^1^ Chemistry Research Laboratory Department of Chemistry University of Oxford 12 Mansfield Road Oxford OX1 3TA UK; ^2^ Department of Biochemistry University of Oxford South Parks Road Oxford OX1 3QU UK; ^3^ Current address: Department of Molecular Medicine Faculty of Medicine University of Malaya, Jalan Universiti 50603 Kuala Lumpur Malaysia; ^4^ Current address: GSK Medicines Research Centre Gunnels Wood Road Stevenage SG1 2NY UK

**Keywords:** hypoxia, hypoxia-inducible factors (HIFs), hydroxylases, 2-oxoglutarate, oxygenases, proteomics

## Abstract

The hypoxia‐inducible factors (HIFs) are key transcription factors in determining cellular responses involving alterations in protein levels in response to limited oxygen availability in animal cells. 2‐Oxoglutarate‐dependent oxygenases play key roles in regulating levels of HIF and its transcriptional activity. We describe MS‐based proteomics studies in which we compared the results of subjecting human breast cancer MCF‐7 cells to hypoxia or treating them with a cell‐penetrating derivative (dimethyl *N*‐oxalylglycine; DMOG) of the stable 2OG analogue *N*‐oxalylglycine. The proteomic results are consistent with reported transcriptomic analyses and support the proposed key roles of 2OG‐dependent HIF prolyl‐ and asparaginyl‐hydroxylases in the hypoxic response. Differences between the data sets for hypoxia and DMOG might reflect context‐dependent effects or HIF‐independent effects of DMOG.

## Graphical abstract

**Coming up for air**: We describe MS‐based proteomics studies in which we compared the results of subjecting human breast cancer MCF‐7 cells to hypoxia or treating them with a cell‐penetrating derivative (dimethyl *N*‐oxalylglycine) of the stable 2OG analogue *N*‐oxalylglycine.

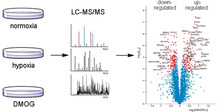

## Introduction

In response to limited oxygen availability, eukaryotic cells adapt by altering the expression of multiple genes in a context‐dependent manner.^1^ The α,β‐hypoxia‐inducible factors (HIFs) are key transcription factors for the hypoxic response in animal cells.^2^ Low oxygen concentrations reduce the activity of the HIF prolyl hydroxylase domain–containing proteins (PHD1–3) and factor inhibiting HIF (FIH), which are human 2‐oxoglutarate (2OG)‐dependent oxygenases that suppress HIF′s transcriptional activity by post‐translational modification of HIF‐α subunits.^3^, ^4^, ^5^, ^6^ As a result, transcriptionally active HIF levels increase, leading to the context‐dependent upregulation of HIF target genes. The hypoxic response is also proposed to be important in many tumours, which are often hypoxic due to poor vascularisation.^7^, ^8^, ^9^ Hypoxic tumour cells have been found to have increased metastatic potential and resistance to radiotherapy and chemotherapy.^10^, ^11^, ^12^ An improved characterisation of gene regulation in response to hypoxia is of interest to further understand the fundamental biology of the hypoxic response, which in turn might help to inform the development of drugs aimed at suppressing or enhancing levels of HIF.^13^, ^14^, ^15^, ^16^, ^17^

Genome‐wide transcription‐profiling studies have been conducted to compare hypoxic and normoxic cells using RNA sequencing and microarray analyses.^18^, ^19^, ^20^, ^21^ There are many mechanisms downstream of transcription that can affect protein concentrations in cells (such as splice variants, transcriptional regulation, post‐translational modifications and protein degradation), and it is well documented that protein abundance does not always correlate with RNA expression levels.^22^, ^23^, ^24^, ^25^, ^26^ Therefore, studies to quantify changes in protein levels are important for a detailed understanding of the cellular hypoxic response.

Several studies have suggested that there is a response at the translational level to hypoxia, in addition to the transcriptional response mediated by HIF.^27^, ^28^, ^29^, ^30^, ^31^ For example, hypoxia has been reported to suppress cap‐mediated translation by sequestering the translational initiation factor eIF4E.^23^, ^24^, ^25^, ^27^ An alternative translational initiation complex containing the hypoxically upregulated HIF‐2α isoform has been identified, which selectively rescues cap‐dependent protein synthesis in hypoxic cells.^30^ It has also been proposed that, in addition to the HIF hydroxylases, other 2OG‐oxygenases might play roles in regulating the hypoxic response, including at the translational level.^32^ OGFOD1 catalyses hydroxylation of a proline residue in a ribosomal protein (RPS23), and has been reported to be associated with changes in translational accuracy.^32^, ^33^ It is anticipated that comparisons of global RNA expression levels with protein levels in response to hypoxia might help reveal the mechanisms, other than transcriptional regulation, that constitute the hypoxic response.

The discovery that the human HIF‐mediated hypoxic response is signalled for by the reduced activity of 2OG‐oxygenases PHD1–3 and FIH has inspired the development of pharmacological methods for stimulating the HIF response.^14^ Dimethyloxalylglycine (DMOG) is a cell‐permeable ester derivative of the near isosteric 2OG analogue *N*‐oxalylglycine (NOG), which inhibits (to different extents) the HIF prolyl and asparaginyl hydroxylases along with other 2OG‐oxygenases.^3^, ^5^, ^34^, ^35^ In a global RNA analysis of DMOG‐ and hypoxia‐treated cells, a good, but imperfect, overall correlation was found between DMOG‐ and hypoxia‐induced gene regulation.^18^, ^19^ However, some genes were found to be regulated by hypoxia, but not apparently by DMOG; it has been suggested that this might be due to non‐2OG‐dependent oxygenase hypoxic regulation. Comparison of protein responses to hypoxia and DMOG could help to identify these regulation mechanisms, and highlight post‐transcriptional differences between DMOG and hypoxic regulation. DMOG and other PHD inhibitors are also of interest for the treatment of ischemic diseases, but the effects of the molecules beyond simulating the hypoxic response, for example, through the inhibition of other 2OG‐oxygenases, are still not established.^14^ Comprehensive transcriptomic and proteomic studies on the effects of 2OG‐oxygenases inhibitors will be of value to inform the design and use of therapeutics targeting the HIF pathway while minimising toxicity. Such considerations would be particularly important in the context of the long‐term use of HIF modulators for the treatment of chronic diseases (such as anaemia), the current target indications of PHD inhibitors in clinical use and development.

To date, the effects of hypoxia and DMOG on protein levels have been studied on small selections of proteins using western blotting, 2D gel electrophoresis and relatively low sensitivity mass spectrometry (MS)‐based proteomics (<1000 proteins quantified).^36^, ^37^, ^38^, ^39^, ^40^ Recent developments in MS‐based proteomic techniques allow the simultaneous quantification of thousands of proteins; however, these methods have not yet been applied to the hypoxic system. We describe a global MS‐based proteomics study of the hypoxic response in human breast cancer MCF‐7 cells comparing the results with DMOG treatment. Protein levels in cells treated with normoxia, hypoxia, or DMOG were compared using triplex dimethyl labelling and LC‐MS/MS analysis.

## Results

**Figure 1:**
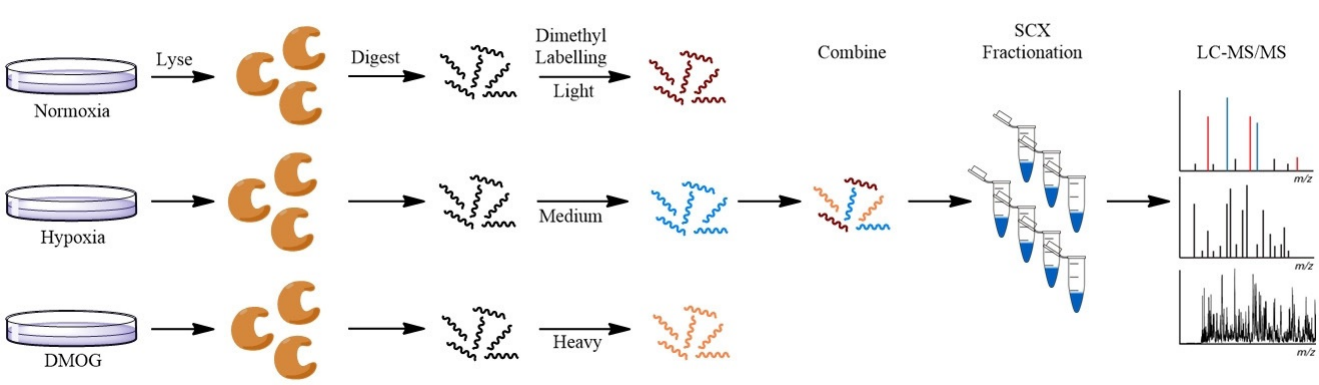
An overview of the protocol for the global comparison of protein levels in cells subjected to normoxia, hypoxia (0.5 % O_2_, 16 h), or treated with DMOG (1 mm, 16 h). Cells were lysed, digested and dimethyl isotope labelled as light (normoxia), medium (hypoxia) or heavy (DMOG). Labelled digests were mixed and separated into seven fractions by using an SCX cartridge. Fractions were analysed by LC‐MS/MS, and protein ratios were calculated based on peptide ratios in the mass spectra (MaxQuant).

Human breast cancer MCF‐7 cells were cultured 1) under normoxia, 2) under hypoxia (0.5 % O_2_) or 3) with inhibitor (1 mm DMOG) for 16 h, in triplicate, before harvesting (Figure 1). The nine cell pellets were lysed in 8 m urea solution, then digested by treatment with dithiothreitol (DTT), iodoacetamide, then Lys‐C and trypsin according to reported protocols.^41^, ^42^ Triplex dimethyl labelling of samples as light (normoxia), medium (hypoxia) and heavy (DMOG treated) was carried out “on column”, as described.^43^ Completion of labelling was confirmed by LC‐MS/MS analysis (1 h gradient, collision induced dissociation (CID)), which indicated >99 % labelling of peptides in all samples. The nine samples were divided into two, and the remaining steps were carried out in technical duplicates. Light, medium and heavy samples were mixed in a ratio of 1:1:1 based on base peak intensities in LC‐MS/MS ion chromatograms to afford six light/medium/heavy mixed samples. The six samples were fractionated by using an SCX‐cartridge into seven fractions, as described in the Supporting Information. The resulting 6×7 fractions were analysed by using an Orbitrap Elite Hybrid Ion Trap‐Orbitrap mass spectrometer with a 4 h LC gradient. Fragmentation of peptides was either by CID or electron transfer dissociation (ETD) according to a data‐dependent decision tree (DDDT)). Analysis of the MS data was performed by using MaxQuant. The 42 LC‐MS/MS runs were annotated in MaxQuant as six experiments, with seven fractions per experiment. Subsequent downstream analysis was performed by using Perseus.

Analysis of the MS data using MaxQuant identified 38 048 peptides and 4560 proteins. Proteins for which a ratio was determined in less than three experiments were removed, to afford 3366, 3348 and 3365 proteins in the hypoxia/normoxia (M/L), DMOG/normoxia (H/L) and DMOG/hypoxia (H/M) datasets, respectively. Analysis of the hypoxia/normoxia data set will be described first; this represents the response to hypoxia, with the subsequent description of the DMOG/normoxia and DMOG/hypoxia data sets, reflecting the response to DMOG as compared to hypoxia.

### Analysis of protein levels in hypoxia versus normoxia

**Figure 2:**
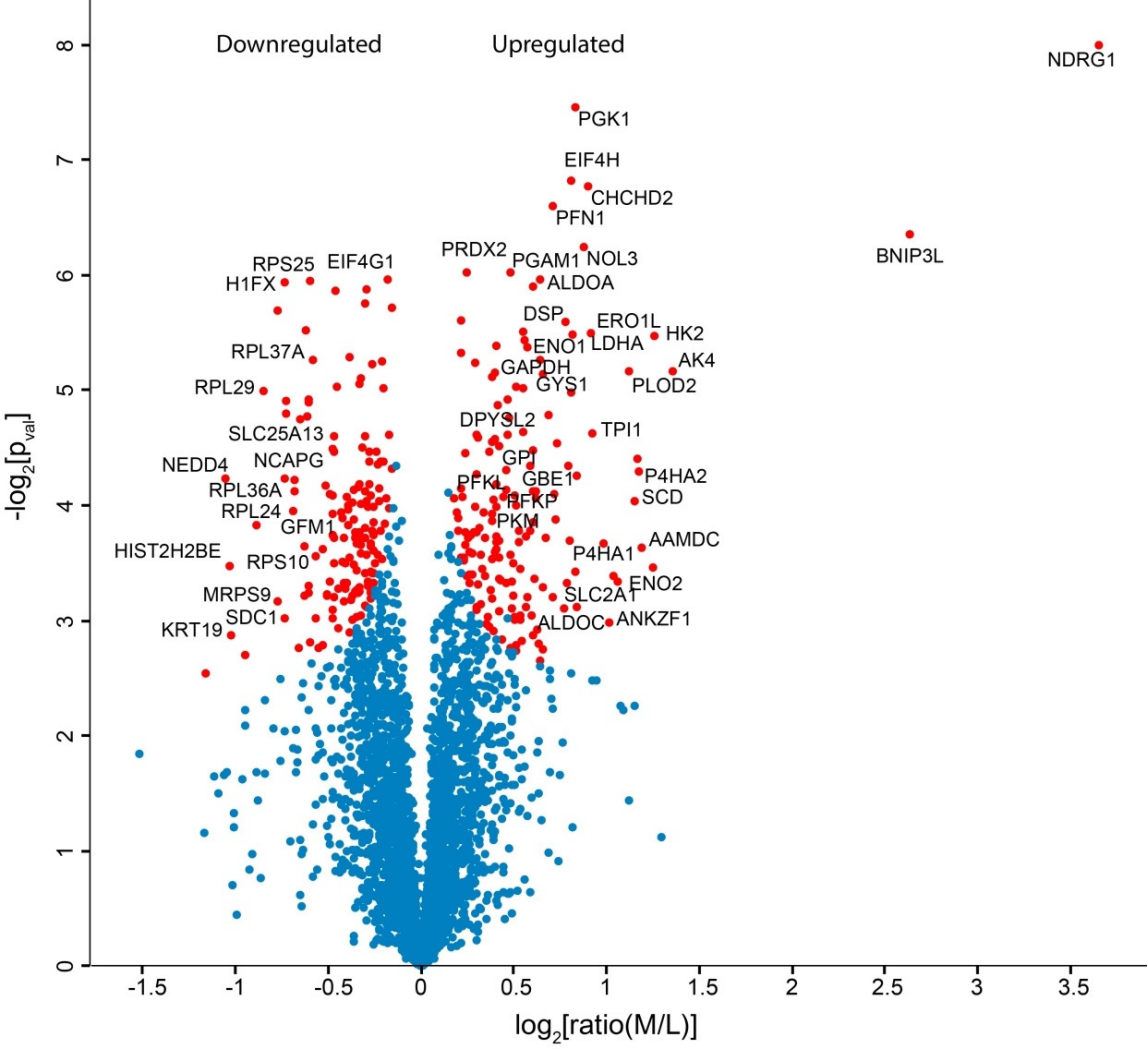
Differential regulation of proteins in hypoxia versus normoxia (M/L). The volcano plot shows the ratio change (*x*‐axis) and non‐zero confidence (*y*‐axis) for each protein. Up‐ and downregulated proteins (red) were identified by using a modified t‐test (Perseus “one sample test”; *s*_0_=0.02; Benjamini–Hochberg FDR<0.05). A selection of upregulated and downregulated proteins are labelled.

Data from the 3366 quantified proteins were visualised using a volcano plot of significance (−log[*p* value]) versus ratio change (log[hypoxia/normoxia]) (Figure 2). For the proteins quantified, the distribution of M/L ratios was found to be relatively narrow, with very few proteins changed by more than twofold (log_2_[ratio]>±1; Figure 2). The two proteins with the largest ratio change were NDRG1 (*N*‐myc downstream‐regulated gene 1 protein; 13‐fold upregulated in hypoxia) and BNIP3L (BCL2/adenovirus E1B 19 kDa protein‐interacting protein 3; sixfold upregulated in hypoxia). These are both known HIF target genes and have previously been shown by western blotting analysis to be upregulated in hypoxia.^44^, ^45^ Up‐ and downregulated proteins were selected by application of a “one‐sample test” to the six replicates (performed in Perseus, *s*_0_=0.02, Benjamini–Hochberg FDR<0.05). This analysis identified 163 upregulated and 154 downregulated proteins (Figure 2, red points, Tables S6 and S7 in the Supporting Information).

### Functional annotation of proteins regulated by hypoxia

The potential functions of proteins that were identified as regulated by hypoxia were studied by analysis of their gene ontology biological pathway (GO‐BP) terms and *Kyoto Encyclopedia of Genes and Genomes* (KEGG) terms. Over‐represented GO‐BP and KEGG terms in the up‐ and downregulated protein sets were compared with the total quantified protein population by using the Fisher test (implemented in Perseus, Benjamini–Hochberg FDR>0.02). Consistent with prior transcriptomic analyses,^46^, ^47^ amongst the upregulated proteins, the KEGG pathways “glycolysis” and “fructose and mannose metabolism” were found to be significantly enriched by nine‐ and six‐fold, respectively (Tables 1 and S1). The most over‐represented GO biological pathway terms also related to glycolysis and catabolic processes. This observation is consistent with prior work^46^, ^47^ and highlights an enhancement of glycolytic pathways in hypoxic conditions as the cell shifts toward less efficient nonoxidative forms of carbon metabolism and ATP production, that is, anaerobic glycolysis.^48^, ^49^ Examples of upregulated glycolytic enzymes include fructose biphosphate aldolases A and C (ALDOA and ALDOC), phosphoglycerate kinase (PGK1) and phosphoglycerate mutase 1 (PGAM1). Lactate dehydrogenase A (LDHA) is also upregulated to divert pyruvate from oxidative breakdown in the mitochondria.^49^

**Table 1:** Over‐represented GO and KEGG terms amongst the assigned upregulated proteins in hypoxia.

KEGG pathway	Fold enrichment
glycolysis/gluconeogenesis	8.7
fructose and mannose metabolism	6.3
**GO biological pathway**	
1. glycolysis	12.1
2. glucose catabolic process	10.0
3. hexose catabolic process	8.5
4. monosaccharide catabolic process	8.1
5. alcohol catabolic process	7.1
6. cellular carbohydrate catabolic process	5.7

Among the downregulated proteins, the KEGG term “ribosome” was significantly over‐represented, possibly reflecting a reduction in protein synthesis under hypoxic conditions to conserve ATP (Tables 2 and S2); this is consistent with the proposal that translation is generally suppressed in hypoxia.^50^ Indeed, 26 of the 154 significantly downregulated proteins were ribosomal (e.g., RPL24, RPL29, RPL36A, RPL37A, RPS25). The most significantly over‐represented GO biological pathway term related to all stages of translation and the transport of proteins to the endoplasmic reticulum.

**Table 2:** Over‐represented GO and KEGG terms amongst the assigned downregulated proteins in hypoxia.

KEGG pathway	Fold enrichment
ribosome	8.0
**GO biological pathway**	
1. translational elongation	8.1
2. translational initiation	7.3
3. viral transcription	8.2
4. translational termination	8.1
5. protein complex disassembly	7.7
6. cellular protein complex disassembly	7.7
7. nuclear‐transcribed mRNA catabolic process, nonsense‐mediated decay	6.8
8. protein targeting to ER	6.9
9. macromolecular complex disassembly	6.9

### Comparison of up‐ and downregulated protein annotations with RNA studies reported in the literature

Many of the proteins observed in this study as being upregulated by hypoxia are reported to be HIF‐1 target genes (e.g., ALDOA, BNIP3L, GRAPDH, GPI, HK2, LDHA, PFKL, SLC2A1, TPI1, ENO1, PGK1).^51^ Elveridge et al. conducted a microarray analysis of changes in RNA levels in response to hypoxia (MCF‐7 cells, 1 % O_2_, 16 h).^19^ They found that 246 transcripts were significantly upregulated and 190 transcripts were significantly downregulated (FDR<0.05). Of the 246 upregulated transcripts, 25 were quantified in the MS‐based proteomics study reported here; 56 % (14/25) of these were also found to be significantly upregulated at the protein level (FDR<0.05, Table 3). Of the 190 downregulated transcripts, 47 were quantified here, but only 9 % (4/47) of these were also found to be significantly downregulated (FDR<0.05, Table 4).^19^ Poor correlation between downregulated transcripts and downregulated proteins in hypoxia has been reported previously.^52^, ^53^, ^54^, ^55^, ^56^ This might result from the longer half‐lives of many proteins and/or the operation of post‐transcriptional regulatory mechanisms (e.g., through translational regulation or protein degradation). Interestingly, the fold changes of the significantly regulated transcripts were larger than those of proteins (Tables 3 and 4, bold names, FDR<0.05).

**Table 3:** Genes found to be significantly upregulated in a microarray analysis of RNA levels (FDR<0.05)^19^ that were also quantified in our LC‐MS/MS analyses.

Names^[a]^	Protein ratio	RNA ratio^19^	Names	Protein ratio	RNA ratio^19^
**NDRG1**	12.6	29.7	ILVBL	1.2	4.1
**ALDOC**	1.7	25.4	**HK2**	2.4	4.1
**ENO2**	2.1	22.5	**NOL3**	1.8	4.1
PGM1	1.9	9.72	NR3C1	1.0	3.7
**BNIP3L**	6.2	9.6	**DPYSL2**	1.2	3.7
**P4HA2**	2.3	7.5	**PFKP**	1.5	3.4
**ERO1L**	1.9	7.0	TRA2A	1.2	3.3
GYS1	1.6	6.3	AK3	1.1	2.9
**P4HA1**	2.0	5.3	CD59	0.8	2.9
**SLC2A1**	1.6	5.2	SCARB1	1.4	2.8
**GBE1**	1.5	5.2	FLNB	1.1	2.8
**PGK1**	1.8	4.9	ITGB4	0.9	2.7
S100A6	1.0	4.7

[a] Genes in bold were significantly upregulated at the protein level (FDR<0.05).

**Table 4:** Genes found to be significantly downregulated in both the microarray analysis of RNA levels,^19^ and in the LC‐MS/MS analysis reported here (FDR<0.05).

Names	Protein ratio	RNA ratio^19^	Names	Protein ratio	RNA ratio^19^
OGDH	−1.1	−3.8	RANGAP1	−1.3	−2.6
DNAJA1	−1.3	−3.6	PUS1	−1.2	−2.6

### Comparison of hypoxia protein regulation with RNA‐sequencing results

**Figure 3:**
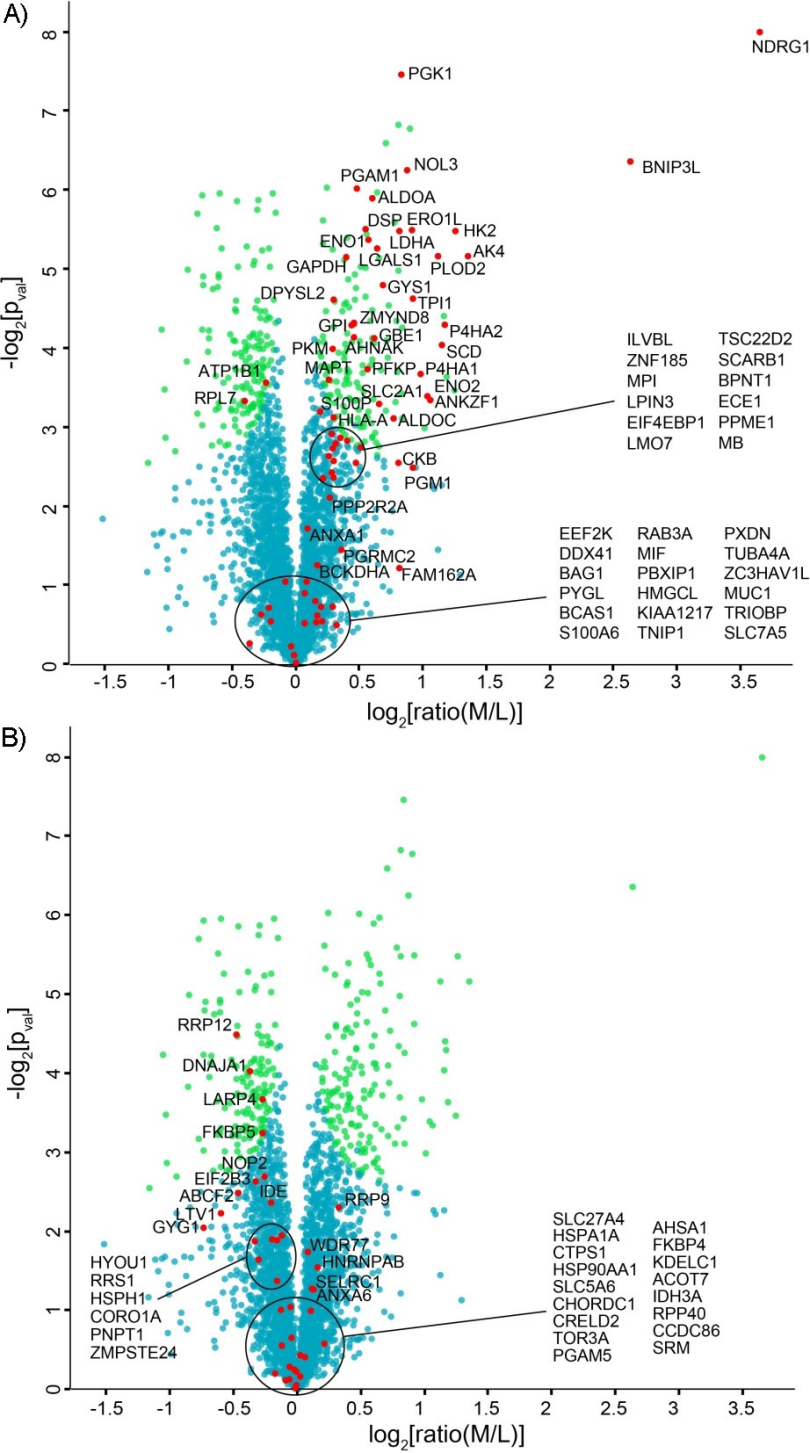
Comparison of protein and mRNA regulation in hypoxia. The volcano plots show the protein response to hypoxia (as in Figure 2). Genes found to be significantly A) up‐ or B) downregulated by hypoxia in an RNA sequencing study are in red. Proteins significantly regulated by hypoxia in the LC‐MS/MS study are in green (FDR<0.05). Good agreement between mRNA and protein ratios was observed for upregulated annotations, but poor agreement was found for downregulated annotations.

An analogous mRNA sequencing study to our proteomic study has been conducted to compare the transcriptional response of MCF‐7 cells treated under the same hypoxic and normoxic conditions.^18^ The mRNA levels of 13 351 genes were studied; 565 and 166 transcripts were observed to be significantly up‐ and downregulated, respectively, after additional filtering to exclude low‐expressed transcripts under normoxic condition (fragments per kilobase of transcripts per million mapped reads, FPKM≥2). The MS‐based proteomics study reported here quantified protein levels for 72 of the 565 transcripts that were upregulated and 38 of the 166 transcripts that were downregulated at the mRNA level (Figure 3, red points). There are correlations among the 72 genes found to be upregulated at the mRNA level, with 68 % of them also being upregulated at the protein level (−log[*p* value]>2, Figure 3 A). There was poorer agreement for the 38 genes that were found to be downregulated at the mRNA level, with only 26 % also downregulated at the protein level (−log[*p* value]>2, Figure 3 B). The most striking difference in the regulation at the mRNA and protein levels was for ATP1B1 and RPL7, which we found to be significantly upregulated at the mRNA level but significantly downregulated at the protein level, thus suggesting possible regulation of them at the translational or protein levels.^18^

### Analysis of protein levels in DMOG versus normoxia

**Figure 4:**
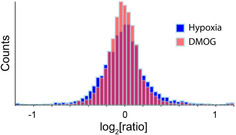
Comparison of protein regulation in hypoxia and DMOG. Overlaid histograms of protein ratios in response to hypoxia (M/L) and DMOG (H/L) indicated that protein levels were more perturbed in response to hypoxia than to DMOG.

Previous RNA studies have indicated that the broad‐spectrum 2OG‐oxygenase inhibitor DMOG (which also inhibits other enzyme types, e.g., isocitrate dehydrogenase)^57^ is an effective mimic of hypoxia in animal cells.^19^ A number of transcripts, however, were found to be regulated by hypoxia, but not by DMOG; this might result from non‐2OG‐oxygenase‐mediated hypoxic regulation (or reflect relatively poor inhibition of a 2OG‐oxygenase by DMOG).^19^, ^58^ The efficacy of DMOG in mimicking hypoxia at the protein level was studied to further investigate non‐2OG‐oxygenase‐dependent regulatory pathways. Comparison of the histograms of ratios obtained in the hypoxia/normoxia (M/L) and DMOG/normoxia (H/L) data sets indicated that, under the tested conditions, proteins were more perturbed in hypoxia than after treatment with DMOG (Figure 4, standard deviation: M/L 0.28, H/L 0.21). This could be due to factors including reduced induction of HIF by DMOG (1 mm) than by hypoxia (0.5 % O_2_), or due to mechanisms of hypoxic regulation that are not induced by DMOG, or a combination of these. Previously, immunoblotting studies indicated that HIF levels induced by DMOG were lower than those induced by hypoxia under the tested conditions.^18^

Proteins significantly up‐ and downregulated in response to DMOG were identified by analysis of the DMOG versus normoxia (H/L) data set by using the statistical “one sample test” in Perseus (*s*_0_=0.01, Benjamini–Hochberg FDR<0.05). Of the 3348 proteins that were quantified in at least three repeats, 40 were found to be upregulated and 39 were downregulated (Figure S1, Tables S8 and S9). This was markedly fewer than were found to be regulated in the hypoxia data set.

Analysis of GO and KEGG terms in the upregulated proteins (Perseus, Fisher test with significantly regulated categorical column, Benjamini–Hochberg FDR<0.02) afforded very similar results to those obtained from the hypoxia data set with glycolytic processes being over‐represented (Table 5). Analysis of the downregulated proteins by this method did not identify any over‐represented terms; this probably reflects the relatively small number of proteins that were annotated as significantly downregulated. A less stringent analysis was conducted to identify GO terms that correlated with high −log_10_[*p* value] among the downregulated proteins (Perseus, Fisher test with −log_10_[*p* value] numerical column, threshold −log_10_[*p* value]>2.5, Benjamini–Hochberg FDR<0.02). This analysis provided very similar results to the analysis of the proteins downregulated in hypoxia, with GO and KEGG terms involving ribosome and translational processes over‐represented (Table 6).

**Table 5:** Over‐represented gene ontology and KEGG terms among the upregulated proteins in DMOG treatment.

KEGG pathway	Fold enrichment
glycolysis/ gluconeogenesis	30.6
fructose and mannose metabolism	25.8
**GO biological pathway**	
1. glycolysis	42.6
2. glucose catabolic process	32.7
3. hexose catabolic process	28.0
4. monosaccharide catabolic process	26.5
5. glucose metabolic process	16.6
6. alcohol catabolic process	21.8

**Table 6:** Over‐represented gene ontology and KEGG terms among downregulated proteins in DMOG treatment (threshold ‐log_10_[*p* value] >2.5).

KEGG pathway	Fold enrichment
	among downregulation
ribosome	6.2
**GO biological pathway**	
1. translational initiation	5.7
2. viral transcription	6.3
3. translational elongation	6.0
4. translational termination	6.2
5. protein complex disassembly	5.9
7. nuclear‐transcribed mRNA catabolic process, nonsense‐mediated decay	5.4
9. RNA catabolic process	4.2
10. protein targeting to ER	5.1

### Comparison of regulation by hypoxia and DMOG

**Figure 5:**
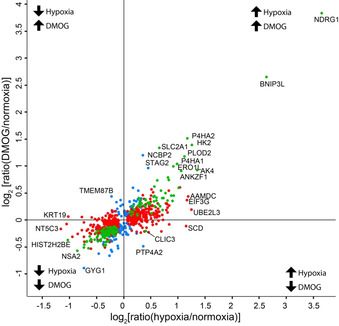
Comparison of protein regulation in hypoxia or DMOG. Protein ratio changes in response to hypoxia and DMOG were plotted for proteins found to be significantly regulated (−log_10_[*p* value]>2.5) by either hypoxia (red), DMOG (blue) or both (green). Most proteins were regulated in the same direction by DMOG and hypoxia, that is, in the top‐right or bottom‐left quadrant. Some proteins appeared to be differentially regulated by hypoxia or DMOG, that is, top‐left and bottom‐right quadrants.

Substantial overlap was found for proteins that were significantly regulated by DMOG or hypoxia, with 75 % (30/40) of the DMOG‐upregulated and 49 % (19/39) of the DMOG‐downregulated genes also up‐/downregulated in hypoxia. The extent of correlation between the two conditions was analysed by direct comparison of the ratio changes. The ratios of proteins that were found to be significantly up‐ and downregulated by hypoxia and/or DMOG were plotted in a scatter plot, with a lower significance threshold {−log_10_[*p* value]>2.5} (Figure 5, significantly regulated by: hypoxia=red, DMOG=blue, both=green). Most proteins were found to be regulated in the same direction by either DMOG or hypoxia (the upper‐right and lower‐left quadrants), and many of these found to be significant in both data sets (green points). The proteins that were found to be regulated in opposite directions were only significant in one data set (blue and red points), with the exception of CLIC3 (chloride intracellular channel protein 3), which was found to be statistically significantly upregulated by hypoxia and downregulated by DMOG (−log_10_[*p* value]>2.5).

### Identification of proteins that are differentially regulated by DMOG or hypoxia

Although most proteins were regulated in the same direction in response to DMOG and hypoxia (relative to normoxia), some proteins were found to be differentially regulated. To further investigate this, the H/M ratios were analysed to identify significantly differentially regulated proteins (Perseus, “one sample test”, *s*_0_=0.01, Benjamini–Hochberg FDR<0.05, Figure S2, Tables S10 and S11). This analysis found 36 proteins to be significantly more abundant in the DMOG‐treated cells and 67 proteins to be significantly more abundant in the hypoxia‐treated cells.

Analysis of GO and KEGG terms in the two differentially regulated protein lists by using Perseus did not identify any significantly over‐represented pathways. A less stringent analysis with *p* value correlation (Perseus, Fisher test based on the numerical column −log[*p* value], threshold −log[*p* value]>2.5) also did not identify any significant terms among the proteins more abundant in hypoxia. Notably, the proteins more abundant in DMOG‐treated cells, however, had over‐represented GO and KEGG terms for the ribosome and translational processes (Table 7).

**Table 7:** Over‐represented GO and KEGG terms among proteins that were more abundant in DMOG‐treated cells compared to hypoxia (threshold *p* value >2.5).

KEGG pathway	Fold enrichment
ribosome	3.4
**GO biological pathway**	
macromolecular complex disassembly	3.5
cellular macromolecular complex subunit organization	2.4
cellular macromolecular complex disassembly	3.5
translational elongation	3.6
RNA catabolic process	2.8
protein complex disassembly	3.5
viral transcription	3.5
protein targeting to ER	3.2
translational termination	3.4
nuclear‐transcribed mRNA catabolic process, nonsense‐mediated decay	3.2

## Discussion

Our MS‐based proteomics study identified 4560 proteins and quantified differences in the abundance of 3366 proteins under hypoxic versus normoxic conditions in MCF‐7 human breast cancer cells. Of the quantified proteins, 163 and 154 were significantly up‐ and downregulated by hypoxia, respectively. It should be noted that these lists are not exhaustive, as only approximately a quarter of the predicted proteome was quantified, and many proteins that were not quantified might be regulated by hypoxia, including regulatory proteins (e.g., transcription factors), which are often present at low abundance. The majority of protein levels were found to change by less than a factor of 2; this is consistent with the ratio changes observed in other global proteomic studies of hypoxia by using MS or 2D gel electrophoresis.^36^, ^37^, ^38^ Immunoblotting studies have reported that some proteins undergo much larger fold changes in hypoxia, for example, HIF, PHD3 and CA9;^19^ however, these proteins were not detected in any of the MS analyses reported here, possibly because they are too low in abundance even after hypoxia‐induced upregulation.

Over‐represented GO and KEGG terms for the proteins found to be upregulated by hypoxia include those related to glycolytic pathways, an observation that is consistent with the well‐established cellular enhancement of glycolytic processes in response to a low‐energy environment such as hypoxia.^59^ Notably, amongst the significantly downregulated proteins, GO and KEGG terms relating to translation and the ribosome were over‐represented. This probably reflects a reduction in protein synthesis during hypoxia in order to preserve energy resources (ATP).^60^, ^61^ Many of the upregulation assignments were consistent with previous literature studies on mRNA and protein levels in response to hypoxia. Several proteins found to be significantly regulated by hypoxia had not previously been reported to be, and these assignments could provide a good starting point for biological studies to develop a detailed understanding of the hypoxic response at the protein level.

The hypoxia‐induced protein regulation was compared with two studies on the mRNA response to hypoxia, one using microarray analysis^19^ and the other using RNA sequencing.^18^ In both comparisons, there was good agreement for the upregulated assignments, but very little overlap in the downregulated assignments. Poor correlation of RNA and protein regulation in response to stimuli, particularly among downregulated genes, has been reported in other studies.^52^, ^53^, ^54^, ^55^, ^56^ This raises the question of to what extent the downregulation of ribosomal proteins under hypoxic conditions is mediated by protein translation or degradation mechanisms rather than by the general reduction in transcription that occurs in hypoxia.

The most striking difference in regulation at the RNA and protein levels was for ATP1B1 (sodium/potassium‐transporting ATPase subunit beta‐1) and RPL7 (ribosomal protein L7), which were assigned as being significantly upregulated at the RNA level but significantly downregulated at the protein level. RPL7 had also been found to be downregulated at the protein level by hypoxia in *Caenorhabditis elegans*,^62^ and ATP1B1 has been reported to be downregulated by hypoxia at the protein and RNA levels in human retinal pigmented epithelial cells.^63^ Further studies are required to validate the changes in protein level observed here; if correct, these observations could offer insight into the post‐transcriptional mechanisms of hypoxic regulation. ATP1B1 is a Na^+^/K^+^‐transporting ATPase that is required for maintaining a normal polarised epithelial phenotype.^63^, ^64^ Decreased ATPase function is associated with epithelial‐to‐mesenchymal transition, which is essential to numerous developmental processes, and in the initiation of metastasis in cancer progression.^65^, ^66^ Hypoxic regulation of ATP1B1 could have implications in cancer pathogenesis.

Protein regulation in response to DMOG was found generally to resemble the response to hypoxia, with substantial overlap between proteins found to be significantly up‐/downregulated in the two data sets. The log[ratio] changes in response to hypoxia were generally larger in magnitude than for DMOG under the tested conditions. Of course, the degree of concord between the data sets might vary with changes in the extent of hypoxia or DMOG concentration, or in different cellular contexts. Immunoblotting studies of the same treatment conditions have indicated that the induction of HIF by DMOG (1 mm) was less pronounced than in hypoxia (0.5 % O_2_), which could cause the observed smaller upregulation of HIF target genes by DMOG.^13^ Additionally, there could be alternative non‐2OG‐oxygenase‐mediated responses to hypoxia that are not activated by DMOG. Some proteins were apparently increased by DMOG treatment but not by hypoxia, potentially reflecting 2OG‐oxygenase‐mediated responses not linked to HIF (e.g., inhibition of JmjC KDMs or ribosome‐modifying 2OG‐oxygenases). However, our focused observations should not be taken as evidence that the regulation of these proteins is HIF independent, as expression of HIF target genes is likely to be limited by other factors in a context‐dependent manner. Note also that DMOG inhibits enzymes other than 2OG‐oxygenases, for example, isocitrate dehydrogenase,^57^ inhibition of which will affect 2OG levels. Analysis of the proteins that were most differentially regulated by hypoxia and DMOG suggests that DMOG is effective at mimicking the enhancement of glycolytic processes by hypoxia, but possibly less effective in suppressing translational processes. This has also been observed at the RNA level in microarray studies.^19^

The differences between the mRNA‐ and protein‐level regulation implied by the combined studies highlight a minimal requirement for both proteomic and transcriptomic studies to ensure an adequate understanding of the hypoxia effects on gene expression. It should be noted that global MS and RNA studies are affected by noise, and any observed differential regulation should be confirmed by alternative techniques including detailed cellular and biochemical studies (in different cell types) before being considered to be validated. It is anticipated that the data sets and analyses reported here could support the initiation of further studies to elucidate the mechanisms and effects of the hypoxic response in different contexts. In addition, application of this MS‐based proteomics approach to the study of selective 2OG‐oxygenase inhibitors (e.g., selective inhibitors of PHD1‐3, FIH or OGFOD1) could help further elucidate the role of particular 2OG‐oxygenases in mediating the hypoxic response.

## Conflict of interest

*The authors declare no conflict of interest*.

## Supporting information

As a service to our authors and readers, this journal provides supporting information supplied by the authors. Such materials are peer reviewed and may be re‐organized for online delivery, but are not copy‐edited or typeset. Technical support issues arising from supporting information (other than missing files) should be addressed to the authors.

Supplementary
