# Supplementary material for: Quantitative MS‐Based Proteomics: Comparing the MCF‐7 Cellular Response to Hypoxia and a 2‐Oxoglutarate Analogue
Source: Chembiochem. 2020 Mar 3;21(11):1647–55. doi: 10.1002/cbic.201900719 (PMC7317498; doi:10.1002/cbic.201900719)
Supplement: Supplementary file 1 — Supplementary [file CBIC-21-1647-s001.pdf]

CHEMBIOCHEM

Supporting Information

Quantitative MS-Based Proteomics: Comparing the MCF-7 Cellular Response to Hypoxia and a 2-Oxoglutarate Analogue

Jacob T. Bush,[a, d] Mun Chiang Chan,[a, c] Shabaz Mohammed,[a, b] and
Christopher J. Schofield*[a]

cbic_201900719_sm_miscellaneous_information.pdf

Author Contributions

J.T.B. carried out the experimental work with assistance in cellular work from M.M.C. S.M. and C.J.S. supervised the work. J.T.B. drafted the manuscript, which was revised by C.J.S. and approved by all authors.

Supplementary Information

Quantitative MS-based proteomics comparing the MCF-7 cellular response to hypoxia and a 2-oxoglutarate analogue

Jacob T. Bush[1Φ], Mun Chiang Chan[1#], Shabaz Mohammed[1,2], and Christopher J. Schofield[1*].

[1] Chemistry Research Laboratory, Department of Chemistry, University of Oxford, 12 Mansfield Road, Oxford, OX1 3TA, United Kingdom.

[2] Department of Biochemistry, University of Oxford, South Parks Rd, Oxford, OX1 3QU, United Kingdom.

# Current address: Department of Molecular Medicine, Faculty of Medicine, University of Malaya, Jalan Universiti, 50603 Kuala Lumpur, Malaysia.

[Φ]Current address: GSK, Medicines Research Centre, Gunnels Wood Road, Stevenage, SG1 2NY, UK

Experiment procedures

Cell culture and treatment

Human breast cancer cells (MCF-7) purchased from the American Type Culture Collection (ATCC) were cultured in Dulbecco’s modified Eagle’s medium (D6546–500ML; Sigma) supplemented with 10% fetal bovine serum (F7524–500ML; Sigma), 2 mM L-glutamine (G7513–100ML; Sigma), 50 units/ml penicillin, and 50 g/ml streptomycin (P0781-100ML; Sigma). Cells were treated with DMOG (1 mM), hypoxia (0.5% O_2) or normoxia for 16 h prior to harvesting. For hypoxia (0.5% O_2) treatment, cells were incubated in an InvivO2 400 hypoxic workstation (Ruskin Technologies, Bridgend, UK).

Cell lysis and digest

Cell pellets were lysed by addition of 8 M urea (1 mL) and sonication (6 x 15 s on, 5 min off). Cell debris was removed by centrifugation (10 min, 14000 rpm) and 200 uL of cell lysate was used for digest. Protein samples in 8 M urea (200 µl) were reduced with DTT (2 µl, 200 mM) at 56 °C for 25 min. The samples were cooled to room temperature and the cysteinyl residues were alkylated by addition of iodoacetamide (4 µl, 36 mg/ml) and incubation in the dark (20 min, room temperature). Dithiothreoitol (2 µl, 200 mM) was added to neutralise the iodoacetamide, before the addition of LysC (1:50, w/w) and incubation at 37°C for 4 h. The samples were diluted with buffer (600 µl, 50 mM ammonium bicarbonate) and trypsin was added (1:50, w/w) followed by further incubation at 37°C for 16 h. The digestion was quenched by the addition of formic acid (10 uL).

Dimethyl labelling

Samples were labelled as light, medium or heavy on-column using isotope-labelled formaldehyde and cyanoborohydride in the following combinations: light – CH_2O, $NaBH_3CN$; medium – CD_2O, $NaBH_3CN$; heavy – $^{13}CD_2O$, $NaBD_3CN$. The labelling reagent contained formaldehyde (0.2% vol/vol) and sodium cyanoborohydride (30 mM) in sodium phosphate buffer (50 mM, pH 7.5). Solvents were: Solvent A – 0.6% acetic acid; Solvent B – 0.6% acetic acid and 80% acetonitrile.

Protocol

A SepPak cartridge (Waters) was washed with acetonitrile (2 mL) and then conditioned with solvent A (2 mL). The digest was loaded onto the SepPak cartridge and washed with solvent A (2 mL). The cartridge was then flushed with labelling reagent (5 mL, over a period of 10 min). The labelling reagent was washed away by elution with solvent A (2 mL), and the labelled peptides were then eluted with solvent B (1 mL). Efficient labelling was checked by LC-MS/MS analysis. The acetonitrile was removed by vacuum centrifuge (1 h, 40 °C) and equal quantities of labelled peptide samples were mixed.

Strong cation exchange (SCX) fractionation

Digests were separated using SCX stage tips (Thermo (SP301), C18, 200 µL tip) as described in the table below. The flow-through during sample loading was collected along with the six elutions of increasing ammonium acetate concentration. The acetonitrile concentration in the seven fractions was reduced by vacuum centrifuge (5 min, 35 °C) before analysis by LC-MS/MS.

	Solvent	Volume (µL)
**Initialise**	1. 1 M NaCl, in solvent C	2 x 30
	2. Solvent C	30
	3. 100 % ACN	4 x 30
	4. 1 M NaCl, in solvent C	30
**Re-equilibrate**	5. Solvent C	3 x 30
**Load sample**	6. 10 µg digest in solvent C	30
	7. solvent C	30
**Elute sample**	8. 15 mM NH_4OAc in solvent C	30
	9. 20 mM NH_4OAc in solvent C	30
	10. 40 mM NH_4OAc in solvent C	30
	11. 70 mM NH_4OAc in solvent C	30
	12. 125 mM NH_4OAc insolvent C	30
	13. 500 mM NH_4OAc in solvent C	30

Solvent C = 20 % acetonitrile, 0.4 % formic acid.

LC-MS/MS analysis and data processing

Digests were analysed by Orbitrap Elite™ (Thermo Fisher Scientific™, DE) connected to a UPLC Proxeon EASY-nLC 1000 and an EASY-Spray nano-electrospray ion source. Peptides were trapped on an Acclaim PepMap® trapping column (100 µm i.d. x 20 mm, 5 µm C18) and separated on an EASY-spray Acclaim PepMap® analytical column (75 µm i.d. x 500 mm, RSLC C18, 2 µm, 100 Å). Solvent A consisted of 0.1% formic acid and solvent B of 0.1% formic acid in acetonitrile. Peptides were separated using a gradient of 7% to 30% solvent B at a flow rate of 200 nL/min. Gradient lengths are specified in the text and were either 1 h or 4 h. Full scan MS spectra were acquired in the Orbitrap (350-1500 m/z, resolution 120,000, AGC target 1e6, maximum injection time 250 ms). CID and ETD spectra were acquired in the Ion Trap (resolution 7500, AGC cation target 3e4, AGC Anion target 2e5, maximum injection time 100 ms). Normalised collision energy for CID and HCD was set to 35% and 32 % respectively. The 20 most intense peaks in the full MS scan were selected for fragmentation using a data-dependent decision tree (DDDT) to select between CID and ETD. In DDDT mode, ETD fragmentation was used for charge states 3, 4 and 5 with m/z less than 750 and for all charge states greater than 5. CID fragmentation was used for all other peptides. Dynamic exclusion was enabled (exclusion list size 75, exclusion duration 5 s).

The raw data files generated were processed using MaxQuant software (Version 1.4.1.2), integrated with the Andromeda search engine. Data were searched against a human database (Swiss-Prot, version 04/13), as well as a list of common contaminants by Andromeda. Protein and PSM false discovery rates (FDR) were set at 0.01. Quantification by dimethyl labelling was performed using

the in-built algorithms in MaxQuant. The minimum number of quantified peptides required per protein was set to 2. The maximum number of missed cleavages was set to be 2. Oxidation (M) and acetylation (*N*-term) were allowed as a variable modification and carbamidomethylation (C) was used as a fixed modification.

Downstream data analysis was performed using Perseus software (Version 1.3.10.4). Protein groups that were quantified in less than three replicates were excluded. Ratio changes and *p* values were calculated using the one-sample test in Perseus. Significant proteins were selected with a false discovery rate < 0.05 (Benjamini-Hochberg method) and s_0 = 0.01 or 0.02 (as specified in the text). The data was visualised in a volcano plot of $\log_2$[ratio change] versus $-\log_{10}$[*p* value]. Gene ontology and KEGG annotations were collected using the Perseus software. Over-represented GO biological pathway and KEGG annotations were identified using the Fisher test (Perseus) by two different methods: i) over-represented terms were identified among proteins selected as significantly up- or down-regulated in the one-sample t-test, (Input column: categorical column, t-test significant; Benjamini-Hochberg FDR threshold = 0.02); ii) over-represented terms were identified among proteins that were up- or down-regulated with high confidence, (Input column: numerical column, $-\log_{10}$[*p* value]; threshold $-\log_{10}$[*p* value] = 2.5; Benjamini-Hochberg FDR threshold = 0.02). Full lists of significantly up- and down-regulated proteins and over-represented GO biological pathway terms are given in tables below.

Supplementary Figures

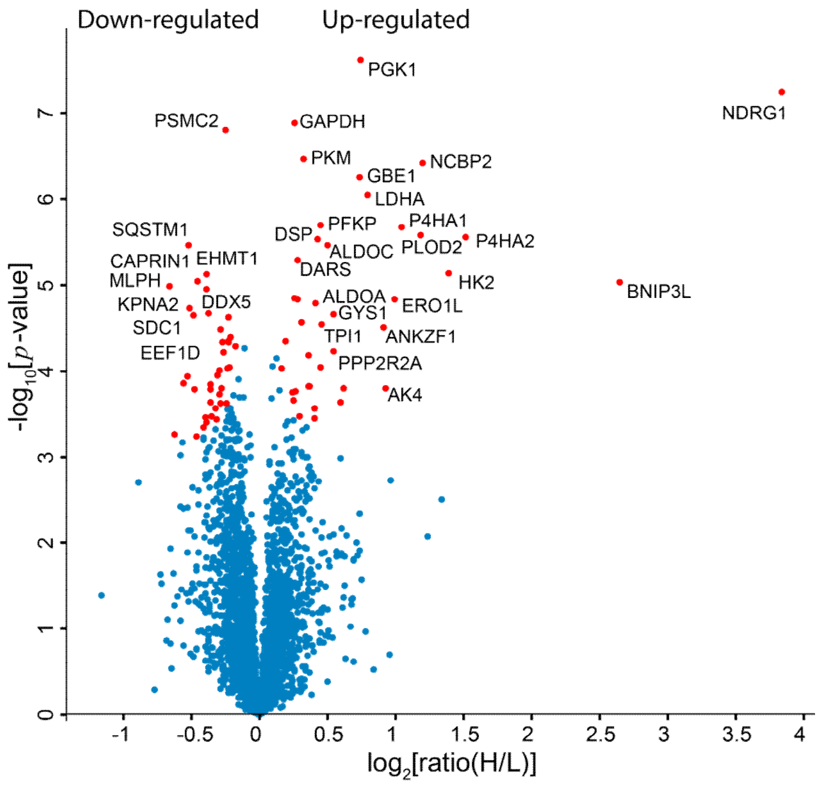

Figure S1. Differential regulation of proteins in DMOG *versus* normoxia (H/L). The volcano plot shows the ratio change (*x*-axis) and non-zero confidence (*y*-axis) for each protein. Significantly regulated proteins (red) were identified using a modified t-test (Perseus 'one sample test'; s_0 = 0.01; Benjamini-Hochberg FDR < 0.05). A selection of significantly regulated proteins are labelled with gene names.

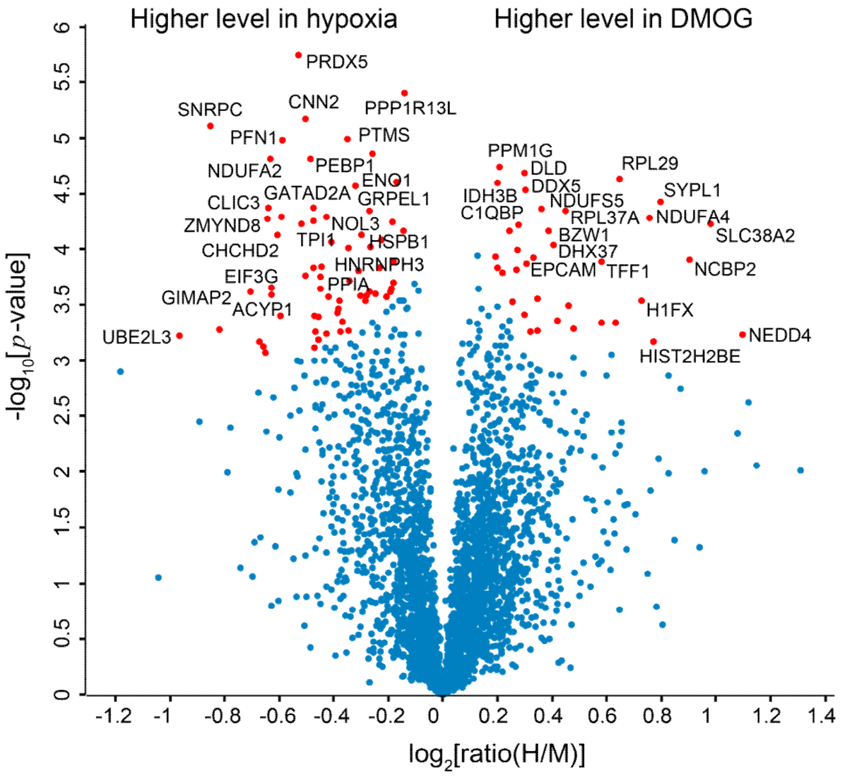

Figure S2. Differential regulation of proteins in DMOG *versus* hypoxia (H/M). A volcano plot to show the ratio change (*x*-axis) and non-zero confidence (*y*-axis) for each protein. Significantly regulated proteins (red) were identified using a modified t-test (Perseus ‘one sample test’; s_0 = 0.01; Benjamini-Hochberg FDR < 0.05). A selection of significantly regulated proteins are labelled with gene names.

Supplementary Tables

Tables S1-5

Full lists of GO biological pathway terms found to be over-represented among the up- and down-regulated proteins using the Fisher test in Persues. The Fisher test was implemented by two methods. First a more stringent test:

i) Over-represented terms were identified among proteins selected as significantly up- or down-regulated in the one-sample t-test, (Input column: categorical column, t-test significant; Benjamini-Hochberg FDR threshold = 0.02);

If this test did not identify any significantly over-represented terms, then a less stringent method was used:

ii) Over-represented terms were identified among proteins that were up-or down-regulated with high confidence, (Input column: numerical column, $-\log_{10}[p \text{ value}]$; threshold $-\log_{10}[p \text{ value}] = 2.5$; Benjamini-Hochberg FDR threshold = 0.02).

Tables S6-11

Full lists of proteins found to be significantly up- and down-regulated in the 3 data sets: medium/light (hypoxia/normoxia), heavy/light (DMOG/normoxia) and heavy/medium (DMOG/hypoxia). Ratio changes and *p* values were calculated using the one-sample test in Perseus. Significant proteins were selected with a false discovery rate < 0.05 (Benjamini-Hochberg method) and s_0 = 0.01 or 0.02 (as specified in the text).

Table S1. Overrepresented GO terms among the hypoxically up-regulated proteins, (Fisher test (i))

Category value	Enrichment factor	Quantified Total (3,366)	Up-regulated (Total 169)	Benj. Hoch. FDR
glycolysis	12.123	23	14	1.27E-09
glucose catabolic process	9.9586	30	15	4.19E-09
hexose catabolic process	8.5359	35	15	4.62E-08
monosaccharide catabolic process	8.0745	37	15	9.09E-08
alcohol catabolic process	7.0817	45	16	1.67E-07
cellular carbohydrate catabolic process	5.7453	52	15	1.42E-05
glucose metabolic process	4.9071	69	17	1.72E-05
carbohydrate catabolic process	5.151	58	15	5.33E-05
gluconeogenesis	8.5359	21	9	0.00018597
hexose metabolic process	3.9371	86	17	0.00035933
small molecule catabolic process	3.3788	112	19	0.00079528
generation of precursor metabolites and energy	2.8639	153	22	0.0017942
monosaccharide metabolic process	3.455	98	17	0.0017947
hexose biosynthetic process	6.4019	28	9	0.0019928
alcohol metabolic process	2.8052	142	20	0.0054854
fructose 1,6-bisphosphate metabolic process	15.934	5	4	0.01133
small molecule metabolic process	1.6271	661	54	0.012585
oxidoreductase activity, acting on paired donors, with incorporation or reduction of molecular oxygen	6.9277	23	8	0.013843
cellular carbohydrate biosynthetic process	4.0572	54	11	0.015247
small molecule biosynthetic process	2.8216	120	17	0.017828

Table S2. Overrepresented GO terms among the hypoxically down-regulated proteins, (Fisher test (i))

Category value	Enrichment factor	Quantified (Total 3,366)	Down-regulated (Total 154)	Benj. Hoch. FDR
translational elongation	8.0802	81	28	1.33E-15
translational initiation	7.289	93	29	3.68E-15
viral transcription	8.2128	74	26	6.18E-15
translational termination	8.1033	75	26	6.84E-15
protein complex disassembly	7.693	79	26	2.02E-14
cellular protein complex disassembly	7.693	79	26	2.43E-14
nuclear-transcribed mRNA catabolic process, nonsense-mediated decay	6.8177	96	28	3.26E-14
protein targeting to ER	6.8601	92	27	5.79E-14
macromolecular complex disassembly	6.8601	92	27	6.27E-14
establishment of protein localization in endoplasmic reticulum	6.8601	92	27	6.84E-14
SRP-dependent cotranslational protein targeting to membrane	6.9354	91	27	6.89E-14
cotranslational protein targeting to membrane	6.8601	92	27	7.52E-14
cellular macromolecular complex disassembly	6.8601	92	27	8.36E-14
nuclear-transcribed mRNA catabolic process	5.5112	123	29	2.19E-12
protein targeting to membrane	5.8984	107	27	3.30E-12
establishment of protein localization to organelle	5.1944	135	30	3.40E-12
mRNA catabolic process	5.3376	127	29	4.46E-12
RNA catabolic process	5.1186	137	30	4.62E-12
cellular component disassembly at cellular level	5.4091	121	28	8.07E-12
cellular component disassembly	5.4091	121	28	8.50E-12
cellular process involved in reproduction	5.049	125	27	1.42E-10
protein targeting	3.9169	185	31	2.60E-09
cellular macromolecular complex subunit organization	3.2365	260	36	1.21E-08
cellular macromolecule catabolic process	3.168	273	37	1.21E-08
reproductive process	3.2307	246	34	4.75E-08
macromolecule catabolic process	2.9315	303	38	5.81E-08
cellular catabolic process	2.3475	468	47	5.43E-07
translation	4.289	109	20	2.96E-06
mRNA metabolic process	2.4908	366	39	3.44E-06
intracellular protein transport	2.7161	284	33	6.72E-06
catabolic process	2.0701	542	48	1.88E-05
macromolecular complex subunit organization	2.3499	378	38	2.39E-05
protein complex subunit organization	2.6641	272	31	2.68E-05
intracellular transport	2.0789	461	41	0.00017936
RNA biosynthetic process	1.9305	448	37	0.0035647
cellular macromolecule biosynthetic process	1.8442	507	40	0.0043304
macromolecule biosynthetic process	1.8333	510	40	0.0048191
establishment of localization in cell	1.8048	531	41	0.0052405
protein transport	1.851	442	35	0.01292
nucleobase-containing compound metabolic process	1.4461	1083	67	0.012922
establishment of protein localization	1.8426	444	35	0.013426
nucleic acid metabolic process	1.5023	918	59	0.01426
RNA metabolic process	1.5583	780	52	0.016975

Table S3. Overrepresented GO terms among the DMOG up-regulated proteins, (Fisher test (i))

Category value	Enrichment factor	Quantified (Total 3,348)	Up-regulated (Total 40)	Benj. Hoch. FDR
glycolysis	42.604	23	12	1.47E-14
glucose catabolic process	32.663	30	12	4.43E-13
hexose catabolic process	27.997	35	12	2.73E-12
monosaccharide catabolic process	26.484	37	12	4.46E-12
glucose metabolic process	16.568	69	14	1.72E-11
alcohol catabolic process	21.776	45	12	4.30E-11
cellular carbohydrate catabolic process	18.844	52	12	2.49E-10
hexose metabolic process	13.293	86	14	2.75E-10
carbohydrate catabolic process	16.895	58	12	7.93E-10
monosaccharide metabolic process	11.666	98	14	1.43E-09
generation of precursor metabolites and energy	8.0057	153	15	4.74E-08
alcohol metabolic process	8.0508	142	14	2.02E-07
gluconeogenesis	27.22	21	7	1.07E-06
cellular carbohydrate metabolic process	7.0569	162	14	1.09E-06
small molecule catabolic process	8.7491	112	12	1.46E-06
cellular carbohydrate biosynthetic process	13.61	54	9	3.22E-06
hexose biosynthetic process	20.415	28	7	8.37E-06
carbohydrate biosynthetic process	11.666	63	9	1.17E-05
carbohydrate metabolic process	5.604	204	14	1.48E-05
fructose 1,6-bisphosphate metabolic process	65.327	5	4	2.95E-05
monosaccharide biosynthetic process	14.657	39	7	7.86E-05
alcohol biosynthetic process	12.426	46	7	0.00024287
fructose metabolic process	36.293	9	4	0.00061916
small molecule metabolic process	2.5982	660	21	0.0011454
small molecule biosynthetic process	6.1759	119	9	0.0019756
heterocycle metabolic process	4.3902	186	10	0.01092
protein tetramerization	10.469	39	5	0.019475

Table S4. Overrepresented GO terms among the DMOG down-regulated proteins (Fisher test (ii))

Category value	Enrichment factor	Quantified (Total 3,348)	Up-regulated (Total 365)	Benj. Hoch. FDR
translational initiation	5.7205	93	58	1.92E-30
viral transcription	6.3217	74	51	7.74E-30
translational elongation	6.0018	81	53	1.16E-29
translational termination	6.2374	75	51	1.46E-29
protein complex disassembly	5.9216	79	51	3.57E-28
cellular protein complex disassembly	5.9216	79	51	4.29E-28
nuclear-transcribed mRNA catabolic process, nonsense-mediated decay	5.3507	96	56	6.65E-28
macromolecular complex disassembly	5.3839	92	54	4.19E-27
cellular macromolecular complex disassembly	5.3839	92	54	4.71E-27
SRP-dependent cotranslational protein targeting to membrane	5.1407	91	51	3.60E-24
RNA catabolic process	4.2181	137	63	4.41E-24
protein targeting to ER	5.0848	92	51	5.21E-24
establishment of protein localization in endoplasmic reticulum	5.0848	92	51	5.61E-24
cotranslational protein targeting to membrane	5.0848	92	51	6.08E-24
mRNA catabolic process	4.3335	127	60	9.34E-24
nuclear-transcribed mRNA catabolic process	4.3999	123	59	9.84E-24
protein targeting to membrane	4.6292	107	54	5.91E-23
cellular component disassembly at cellular level	4.2452	121	56	1.62E-21
cellular component disassembly	4.2452	121	56	1.71E-21
cellular process involved in reproduction	4.1093	125	56	1.15E-20
establishment of protein localization to organelle	3.8049	135	56	1.09E-18
mRNA metabolic process	2.4191	364	96	1.11E-16
cellular macromolecule catabolic process	2.6978	272	80	1.62E-16
protein targeting	3.0245	185	61	1.07E-14
macromolecule catabolic process	2.4906	302	82	1.12E-14
cellular macromolecular complex subunit organization	2.5598	258	72	2.69E-13
Translation	3.5344	109	42	2.55E-12
reproductive process	2.5187	244	67	7.01E-12
RNA biosynthetic process	1.9905	447	97	6.42E-11
RNA metabolic process	1.6742	778	142	8.89E-11
cellular macromolecule biosynthetic process	1.8997	507	105	1.16E-10
protein complex subunit organization	2.3355	271	69	1.36E-10
macromolecule biosynthetic process	1.8885	510	105	1.63E-10
nucleobase-containing compound metabolic process	1.4877	1079	175	2.43E-09
nucleic acid metabolic process	1.5455	914	154	4.95E-09
intracellular protein transport	2.1716	283	67	9.70E-09

cellular catabolic process	1.8463	467	94	1.31E-08
macromolecular complex subunit organization	1.9516	376	80	2.86E-08
nitrogen compound metabolic process	1.3912	1200	182	3.28E-07
cellular macromolecule metabolic process	1.3444	1385	203	3.30E-07
cellular nitrogen compound metabolic process	1.3898	1188	180	4.64E-07
cellular biosynthetic process	1.5078	803	132	1.47E-06
catabolic process	1.6446	541	97	3.62E-06
macromolecule metabolic process	1.2987	1455	206	5.74E-06
biosynthetic process	1.4716	829	133	5.83E-06
intracellular transport	1.6587	459	83	3.01E-05
establishment of protein localization	1.5979	442	77	0.0003793
protein transport	1.5844	440	76	0.00059809
primary metabolic process	1.1883	1814	235	0.00093524
ribonucleoprotein complex biogenesis	3.7264	32	13	0.0012961
establishment of localization in cell	1.494	528	86	0.0013343
RNA processing	1.673	318	58	0.0015948
cellular component biogenesis at cellular level	3.5072	34	13	0.0026096
rRNA processing	2.6812	65	19	0.0029373
cellular protein metabolic process	1.4079	645	99	0.0030611
rRNA metabolic process	2.6406	66	19	0.0035582
cellular metabolic process	1.1619	1871	237	0.0049159
cellular component organization at cellular level	1.3364	755	110	0.009041
cellular component organization or biogenesis at cellular level	1.3206	771	111	0.013295
cellular component biogenesis	2.9811	40	13	0.01523

Table S5. Overrepresented GO terms among the proteins that were more abundant in DMOG than hypoxia treatment (Fisher test (ii))

Category value	Enrichment factor	Quantified (Total 3,365)	Up-regulated (Total 196)	Benj. Hoch. FDR
macromolecular complex disassembly	3.5456	92	19	0.0012692
cellular macromolecular complex subunit organization	2.3681	261	36	0.0018943
cellular macromolecular complex disassembly	3.5456	92	19	0.0019039
translational elongation	3.6032	81	17	0.0029809
RNA catabolic process	2.757	137	22	0.00435
protein complex disassembly	3.4771	79	16	0.0048317
viral transcription	3.4801	74	15	0.0049634
protein targeting to ER	3.1724	92	17	0.0050403
translational termination	3.4337	75	15	0.0051733
nuclear-transcribed mRNA catabolic process, nonsense-mediated decay	3.2191	96	18	0.0052769
nuclear-transcribed mRNA catabolic process	2.7916	123	20	0.0053593
establishment of protein localization in endoplasmic reticulum	3.1724	92	17	0.0054281
cellular protein complex disassembly	3.4771	79	16	0.005522
macromolecular complex subunit organization	1.9026	379	42	0.0055539
cotranslational protein targeting to membrane	3.1724	92	17	0.0058804
SRP-dependent cotranslational protein targeting to membrane	3.2073	91	17	0.0060853
cellular process involved in reproduction	2.8843	125	21	0.0062507
mRNA catabolic process	2.7037	127	20	0.0076302
cellular component disassembly at cellular level	2.6959	121	19	0.011406
cellular component disassembly	2.6959	121	19	0.012006

Table S6. Significantly up-regulated proteins in hypoxia

Gene names	log[ratio(M/L)]	-log[*p* value]	Protein names
NDRG1	3.65196	7.99845	Protein NDRG1
BNIP3L	2.63296	6.35307	BCL2/adenovirus E1B 19 kDa protein-interacting protein 3-like
AK4	1.35333	5.15997	GTP:AMP phosphotransferase AK4, mitochondrial
HK2	1.26109	5.47294	Hexokinase-2
UBE2L3	1.24692	3.45614	Ubiquitin-conjugating enzyme E2 L3
AAMDC	1.18796	3.63648	Mth938 domain-containing protein
P4HA2	1.17057	4.28981	Prolyl 4-hydroxylase subunit alpha-2
EIF3G	1.16299	4.40437	Eukaryotic translation initiation factor 3 subunit G
SCD	1.15469	4.0403	Acyl-CoA desaturase
PLOD2	1.12049	5.15946	Procollagen-lysine,2-oxoglutarate 5-dioxygenase 2
ANKZF1	1.06332	3.33804	Ankyrin repeat and zinc finger domain-containing protein 1
ENO2	1.03939	3.38698	Gamma-enolase;Enolase
LLPH	1.01402	2.98788	Protein LLP homolog
P4HA1	0.98128	3.66622	Prolyl 4-hydroxylase subunit alpha-1
TPI1	0.920272	4.61824	Triosephosphate isomerase
ERO1L	0.918188	5.48883	ERO1-like protein alpha
CHCHD2	0.900567	6.77136	Coiled-coil-helix-coiled-coil-helix domain-containing protein 2,
NOL3	0.875489	6.24179	Nucleolar protein 3
AGR2	0.842837	4.25207	Anterior gradient protein 2 homolog
DNPH1	0.839983	3.11157	2-deoxynucleoside 5-phosphate N-hydrolase 1
ATP5J	0.833776	3.41939	ATP synthase-coupling factor 6, mitochondrial
PGK1	0.833283	7.45193	Phosphoglycerate kinase 1;Phosphoglycerate kinase
LDHA	0.813528	5.48195	L-lactate dehydrogenase A chain;L-lactate dehydrogenase
EIF4H	0.81256	6.81823	Eukaryotic translation initiation factor 4H
TIMM13	0.805577	4.97481	Mitochondrial import inner membrane translocase subunit Tim13
SNRPC	0.801666	3.69102	U1 small nuclear ribonucleoprotein C
CSTB	0.796485	4.34471	Cystatin-B
ENY2	0.788372	3.31999	Enhancer of yellow 2 transcription factor homolog
PARK7	0.780376	5.59054	Protein DJ-1
ALDOC	0.768463	3.10939	Fructose-bisphosphate aldolase C;Fructose-bisphosphate aldolase
TAGLN2	0.730479	4.54231	Transgelin-2
PCMT1	0.724701	3.87718	Protein-L-isoaspartate(D-aspartate) O-methyltransferase;
TXNDC17	0.714448	4.0955	Thioredoxin domain-containing protein 17
PNPO	0.713759	3.20837	Pyridoxine-5-phosphate oxidase
PFN1	0.710351	6.59173	Profilin-1
GYS1	0.688817	4.78784	Glycogen [starch] synthase, muscle
C12orf57	0.672509	3.72088	Protein C10
SLC2A1	0.659563	3.28882	Solute carrier family 2, facilitated glucose transporter member 1
RPS21	0.658469	2.75543	40S ribosomal protein S21
SRSF2	0.656756	5.13916	Serine/arginine-rich splicing factor 2
ACYP1	0.644127	2.64898	Acylphosphatase-1;Acylphosphatase
LGALS1	0.643793	5.25564	Galectin-1
SH3BGRL	0.643285	5.96192	SH3 domain-binding glutamic acid-rich-like protein
SNAPIN	0.631525	2.80001	SNARE-associated protein Snapin
SRP9	0.62817	2.92398	Signal recognition particle 9 kDa protein
PRDX5	0.62184	4.06395	Peroxiredoxin-5, mitochondrial
GBE1	0.615707	4.12226	1,4-alpha-glucan-branching enzyme
SCP2	0.614749	3.35719	Non-specific lipid-transfer protein
PEBP1	0.607268	4.47641	Phosphatidylethanolamine-binding protein 1
ADI1	0.606947	2.87806	1,2-dihydroxy-3-keto-5-methylthiopentene dioxygenase
COA6	0.605263	3.85551	Cytochrome c oxidase assembly factor 6 homolog
ALDOA	0.603532	5.89674	Fructose-bisphosphate aldolase A;Fructose-bisphosphate aldolase
HSPE1	0.602965	2.86669	10 kDa heat shock protein, mitochondrial
CRKL	0.602738	4.12533	Crk-like protein
HAGH	0.596899	3.04148	Hydroxyacylglutathione hydrolase, mitochondrial
TSC22D4	0.589003	3.78279	TSC22 domain family protein 4
PPIA	0.587383	4.33937	Peptidyl-prolyl cis-trans isomerase A;Peptidyl-prolyl cis-trans isomerase
ENO1	0.574239	5.36884	Alpha-enolase
DDAH2	0.57318	3.19758	N(G),N(G)-dimethylarginine dimethylaminohydrolase 2
PFKP	0.56626	3.72603	6-phosphofructokinase type C
POLR2H	0.56215	3.11669	DNA-directed RNA polymerases I, II, and III subunit RPABC3
CFDP1	0.558979	5.43685	Craniofacial development protein 1
HNRNPH1	0.552244	5.01195	Heterogeneous nuclear ribonucleoprotein H
DSP	0.549721	5.50152	Desmoplakin
BLVRB	0.547324	4.64201	Flavin reductase (NADPH)
DDAH1	0.542447	2.81822	N(G),N(G)-dimethylarginine dimethylaminohydrolase 1
TAX1BP3	0.535532	3.44411	Tax1-binding protein 3
EIF1AD	0.532409	3.04925	Probable RNA-binding protein EIF1AD
PPIE	0.531492	3.00222	Peptidyl-prolyl cis-trans isomerase E;Peptidyl-prolyl cis-trans isomerase
HNRNPH3	0.527507	3.685	Heterogeneous nuclear ribonucleoprotein H3
DBI	0.525582	3.77324	Acyl-CoA-binding protein
TSC22D2	0.512409	2.73938	TSC22 domain family protein 2
FKBP3	0.511302	5.02923	Peptidyl-prolyl cis-trans isomerase FKBP3
UQCR10	0.509929	2.78106	Cytochrome b-c1 complex subunit 9

SRSF1	0.509506	4.00061	Serine/arginine-rich splicing factor 1
LGALS3	0.505576	3.03343	Galectin-3
SRSF9	0.505291	4.08884	Serine/arginine-rich splicing factor 9
TCEAL3	0.50256	3.0031	Transcription elongation factor A protein-like 3
CCS	0.500711	3.21768	Copper chaperone for superoxide dismutase
SRI	0.496112	3.4943	Sorcin
SRSF7	0.489882	3.34224	Serine/arginine-rich splicing factor 7
PTGES3	0.488217	3.10874	Prostaglandin E synthase 3
PGAM1	0.48428	6.02003	Phosphoglycerate mutase 1
PPP1R13L	0.484269	3.5643	RelA-associated inhibitor
SH3BGRL3	0.478921	2.76702	SH3 domain-binding glutamic acid-rich-like protein 3
AK2	0.473363	4.76082	Adenylate kinase 2, mitochondrial
MTPN	0.470502	4.92206	Myotrophin
ARL3	0.463941	4.60945	ADP-ribosylation factor-like protein 3
GPI	0.462835	4.31085	Glucose-6-phosphate isomerase
ZMYND8	0.462729	4.31106	Protein kinase C-binding protein 1
UBL4A	0.461866	3.32222	Ubiquitin-like protein 4A
AHNAK	0.456851	4.13795	Neuroblast differentiation-associated protein AHNAK
PDIA3	0.443292	4.07006	Thioredoxin
MRPS36	0.438607	2.83353	28S ribosomal protein S36, mitochondrial
MSI2	0.425086	3.34897	RNA-binding protein Musashi homolog 2
ARPC5L	0.424042	3.36054	Actin-related protein 2/3 complex subunit 5-like protein
HIBADH	0.423448	3.5435	3-hydroxyisobutyrate dehydrogenase, mitochondrial
RBM4	0.423285	4.50986	RNA-binding protein 4
FAHD2A	0.420598	3.08455	Fumarylacetoacetate hydrolase domain-containing protein 2A/2B
NUDCD2	0.417509	3.69753	NudC domain-containing protein 2
GRB2	0.415334	4.87283	Growth factor receptor-bound protein 2
U2AF2	0.408406	3.67883	Splicing factor U2AF 65 kDa subunit
PFN2	0.408391	4.18302	Profilin;Profilin-2
G3BP1	0.407841	3.98975	Ras GTPase-activating protein-binding protein 1
CPOX	0.40647	3.61593	Coproporphyrinogen-III oxidase, mitochondrial
CLIC3	0.403987	3.73532	Chloride intracellular channel protein 3
ERP29	0.403785	5.38811	Endoplasmic reticulum resident protein 29
ALKBH5	0.395582	3.60093	RNA demethylase ALKBH5
GAPDH	0.39526	5.15031	Glyceraldehyde-3-phosphate dehydrogenase
ANP32B	0.394646	4.57557	Acidic leucine-rich nuclear phosphoprotein 32 family member B
GCA	0.392545	2.9056	Grancalcin
WRNIP1	0.38774	4.04479	ATPase WRNIP1
GSN	0.38578	5.11153	Gelsolin
CFL1	0.385105	3.18924	Cofilin-1
HSPB1	0.382678	3.86699	Heat shock protein beta-1
PPIH	0.381471	4.55271	Peptidyl-prolyl cis-trans isomerase H
HNRNPA3	0.380318	3.92778	Heterogeneous nuclear ribonucleoprotein A3
HNRNPA2B1	0.379509	3.53655	Heterogeneous nuclear ribonucleoproteins A2/B1
RSBN1	0.37241	3.266	Round spermatid basic protein 1
NME2	0.368963	2.94892	Nucleoside diphosphate kinase
PDIA3	0.364618	4.46279	Protein disulfide-isomerase A3
ACO1;IRP1	0.360064	3.03698	Cytoplasmic aconitate hydratase
RBBP4	0.349385	2.97191	Histone-binding protein RBBP4
CYB5A	0.347139	3.71363	Cytochrome b5
NDUFA7	0.34295	3.39031	NADH dehydrogenase [ubiquinone] 1 alpha subcomplex subunit 7
Q14980-2	0.335627	3.93526	
CDC42EP4	0.329591	3.45069	Cdc42 effector protein 4
EIF1;EIF1B	0.320449	3.1365	Eukaryotic translation initiation factor 1;Eukaryotic translation initiation factor 1b
NHP2L1	0.320164	3.56632	NHP2-like protein 1
EIF5A	0.315781	3.80223	Eukaryotic translation initiation factor 5A-1
LMNA	0.308018	4.58411	Prelamin-A/C;Lamin-A/C
TMOD3	0.306271	3.30748	Tropomodulin-3
DPYSL2	0.303251	4.60881	Dihydropyrimidinase-related protein 2
BANF1	0.302734	3.76524	Barrier-to-autointegration factor
GLO1	0.298224	4.27299	Lactoylglutathione lyase
HLA-A	0.297448	3.12022	HLA class I histocompatibility antigen, A-2 alpha chain
VBP1	0.297363	3.09055	Prefoldin subunit 3
PPA1	0.295327	5.24151	Inorganic pyrophosphatase
PKM	0.293757	3.98781	Pyruvate kinase isozymes M1/M2
CRK	0.282237	3.76311	Adapter molecule crk
RBM8A	0.279276	3.39552	RNA-binding protein 8A
MOCS2	0.274868	3.58213	Molybdopterin synthase sulfur carrier subunit
PPP1R14B	0.271638	3.74589	Protein phosphatase 1 regulatory subunit 14B
NUTF2	0.265205	3.32223	Nuclear transport factor 2
MAPT	0.26205	3.59435	Microtubule-associated protein tau;Microtubule-associated protein
FHL1	0.260972	3.396	Four and a half LIM domains protein 1
S100A10	0.253767	3.72036	Protein S100-A10
PRDX2	0.245796	6.02486	Peroxiredoxin-2
HDDC2	0.244456	3.39026	HD domain-containing protein 2
WASL	0.242384	4.45759	Neural Wiskott-Aldrich syndrome protein
BOLA2	0.238965	3.65594	BolA-like protein 2

ACOT13	0.235889	3.7615	Acyl-coenzyme A thioesterase 13
ANXA5	0.230329	3.517	Annexin A5;Annexin
RBBP7	0.228632	3.54983	Histone-binding protein RBBP7
TNKS1BP1	0.223987	4.0718	182 kDa tankyrase-1-binding protein
TFG	0.218569	5.32319	Protein TFG
CRABP1	0.216829	4.15001	Cellular retinoic acid-binding protein 1
PTPRF	0.213503	3.55126	Receptor-type tyrosine-protein phosphatase F
DNM2	0.212535	5.60966	Dynamin-2
HPRT1	0.201706	3.77289	Hypoxanthine-guanine phosphoribosyltransferase
PFKL	0.198954	3.88529	6-phosphofructokinase, liver type
SERPINB6	0.19519	3.94002	Serpin B6
CLTA	0.180532	4.0666	Clathrin light chain A

Table S7. Significantly down-regulated proteins in hypoxia

Gene names	log[ratio(M/L)]	-log[*p* value]	Protein names
NT5C3	-1.16216	2.54424	Cytosolic 5-nucleotidase 3
NEDD4	-1.05499	4.23397	E3 ubiquitin-protein ligase;E3 ubiquitin-protein ligase NEDD4
HIST2H2BE	-1.03323	3.46828	Histone H2B type 2-E;
KRT19	-1.02303	2.8661	
ASAP2	-0.945135	2.69507	ANK repeat and PH domain-containing protein 2
NSA2	-0.855152	3.82965	Ribosome biogenesis protein NSA2 homolog
RPL29	-0.851963	4.9875	60S ribosomal protein L29
MRPS9	-0.771316	3.16269	28S ribosomal protein S9, mitochondrial
NDUFA4	-0.770441	5.69181	NADH dehydrogenase [ubiquinone] 1 alpha subcomplex subunit 4
NCAPG	-0.73783	4.23369	Condensin complex subunit 3
H1FX	-0.735342	5.93058	Histone H1x
SDC1	-0.734635	3.01885	Syndecan-1
SLC25A13	-0.729269	4.79006	Calcium-binding mitochondrial carrier protein Aralar2
KPNA2	-0.723921	4.90312	Importin subunit alpha-2
GLTSCR2	-0.687787	3.95427	Glioma tumor suppressor candidate region gene 2 protein
RPL24	-0.684391	4.12752	60S ribosomal protein L24
RPL36A	-0.678094	4.22527	60S ribosomal protein L36a;60S ribosomal protein L36a-like
H1F0	-0.661647	2.76394	Histone H1.0
SQSTM1	-0.652903	4.74081	Sequestosome-1
RPS10	-0.627782	3.64231	40S ribosomal protein S10
KIAA0391	-0.624249	3.21129	Mitochondrial ribonuclease P protein 3
DDX5	-0.618632	5.51826	Probable ATP-dependent RNA helicase DDX5
CHTOP	-0.610075	4.76562	Chromatin target of PRMT1 protein
RPL35	-0.605984	3.24207	60S ribosomal protein L35
RHOT2	-0.60385	4.89251	Mitochondrial Rho GTPase 2
RPL13	-0.603732	4.92217	60S ribosomal protein L13
UBA3	-0.601652	3.30406	NEDD8-activating enzyme E1 catalytic subunit
RPS25	-0.599134	5.95183	40S ribosomal protein S25
BRIP1	-0.595535	2.81541	Fanconi anemia group J protein
RPL37A	-0.579386	5.25953	60S ribosomal protein L37a
GNL2	-0.570188	3.56133	Nucleolar GTP-binding protein 2
RPS6	-0.567262	3.01453	40S ribosomal protein S6
PPIG	-0.55108	2.75924	Peptidyl-prolyl cis-trans isomerase G;Peptidyl-prolyl cis-trans isomerase
TFF1	-0.531505	3.61957	Trefoil factor 1
DAP3	-0.527303	2.78126	28S ribosomal protein S29, mitochondrial
RPL19	-0.514721	4.17018	Ribosomal protein L19;60S ribosomal protein L19
RPS16	-0.509607	3.21052	40S ribosomal protein S16
NSUN5	-0.504189	3.23029	Putative methyltransferase NSUN5
DDX21	-0.489988	4.09892	Nucleolar RNA helicase 2
ATP1B3	-0.488539	3.33175	Sodium/potassium-transporting ATPase subunit beta-3
RRP12	-0.478211	4.49207	RRP12-like protein
RPS14	-0.477643	3.92211	40S ribosomal protein S14
CSDE1	-0.476915	4.08086	Cold shock domain-containing protein E1
RPS23	-0.472412	3.7414	40S ribosomal protein S23
HECTD1	-0.472308	3.01426	E3 ubiquitin-protein ligase HECTD1
RPL28	-0.4722	3.08972	60S ribosomal protein L28
SLC35B2	-0.470465	4.60187	Adenosine 3-phospho 5-phosphosulfate transporter 1
RPL8	-0.469149	3.49576	60S ribosomal protein L8
RPL17	-0.468587	3.71469	60S ribosomal protein L17
GTPBP4	-0.467384	4.46159	Nucleolar GTP-binding protein 1
KLHDC4	-0.464773	3.1998	Kelch domain-containing protein 4
KIAA1324	-0.46322	5.85638	UPF0577 protein KIAA1324
PRPF3	-0.455495	5.03174	U4/U6 small nuclear ribonucleoprotein Prp3
RRP1	-0.447902	2.9311	Ribosomal RNA processing protein 1 homolog A
RPL23A	-0.442399	3.27941	60S ribosomal protein L23a
OXA1L	-0.430845	3.94152	Mitochondrial inner membrane protein OXA1L
VRK1	-0.425471	3.50953	Serine/threonine-protein kinase VRK1
PSMD6	-0.423276	3.89314	26S proteasome non-ATPase regulatory subunit 6
NDUFB11	-0.41778	3.16687	NADH dehydrogenase [ubiquinone] 1 beta subcomplex subunit 11
RPS11	-0.416354	3.71532	40S ribosomal protein S11
C19orf52	-0.411293	3.32154	Uncharacterized protein C19orf52

SLC1A5	-0.405495	3.49109	Neutral amino acid transporter B(0)
RPL32	-0.405211	3.27066	60S ribosomal protein L32
RPL7	-0.396956	3.32211	60S ribosomal protein L7
RHOC;RHOA	-0.396392	4.07175	Rho-related GTP-binding protein RhoC
USMG5	-0.395705	3.83231	Up-regulated during skeletal muscle growth protein 5
FUNDC2	-0.395233	4.00474	FUN14 domain-containing protein 2
EIF3E	-0.389998	3.96586	Eukaryotic translation initiation factor 3 subunit E
EIF3C	-0.387536	5.28451	Eukaryotic translation initiation factor 3 subunit C
RPS3A	-0.386283	3.54701	40S ribosomal protein S3a
RPL6	-0.386239	2.9013	60S ribosomal protein L6
CLUH	-0.374935	3.18209	Clustered mitochondria protein homolog
FTSJD2	-0.370662	3.2168	Cap-specific mRNA (nucleoside-2-O-)-methyltransferase 1
DNAJA1	-0.370316	4.02898	DnaJ homolog subfamily A member 1
ATAD2	-0.369324	3.00501	ATPase family AAA domain-containing protein 2
H2AFY	-0.363787	3.48838	Core histone macro-H2A.1;Histone H2A
EHD4	-0.363082	3.87493	EH domain-containing protein 4
EEF1D	-0.360425	4.1365	
CRNKL1	-0.355466	3.71204	Crooked neck-like protein 1
METAP2	-0.352992	3.25789	Methionine aminopeptidase 2;Methionine aminopeptidase
RPL22	-0.350723	3.76548	60S ribosomal protein L22
MPG	-0.347794	3.25797	DNA-3-methyladenine glycosylase
RPL27A	-0.346766	3.43333	60S ribosomal protein L27a
GFM1	-0.344212	3.66484	Elongation factor G, mitochondrial
PPP1CC	-0.343191	3.03009	Serine/threonine-protein phosphatase
RPS2	-0.342736	3.2377	40S ribosomal protein S2
SRP19	-0.34145	4.15825	Signal recognition particle 19 kDa protein
NRD1	-0.335245	3.76212	Nardilysin
RANGAP1	-0.334623	4.18418	Ran GTPase-activating protein 1
RPS27	-0.333847	3.28481	40S ribosomal protein S27
HMGCS1	-0.332432	5.04961	Hydroxymethylglutaryl-CoA synthase, cytoplasmic
MTCH2	-0.330829	3.28432	Mitochondrial carrier homolog 2
EIF3K	-0.329107	3.25062	Eukaryotic translation initiation factor 3 subunit K
RANBP2	-0.327128	4.01646	E3 SUMO-protein ligase RanBP2
MCM7	-0.324114	5.10025	DNA replication licensing factor MCM7
DDX3X	-0.322473	3.66168	ATP-dependent RNA helicase DDX3X
HNRNPH1	-0.321931	3.04687	
BSG	-0.320388	3.58415	Basigin
GFRA1	-0.314698	3.71585	GDNF family receptor alpha-1
EPCAM	-0.313491	4.49768	Epithelial cell adhesion molecule
PREX1	-0.304197	4.59748	Phosphatidylinositol 3,4,5-trisphosphate-dependent Rac exchanger 1 protein
DDX42	-0.301089	3.80427	ATP-dependent RNA helicase DDX42
PPM1G	-0.300719	5.75101	Protein phosphatase 1G
GNL3	-0.29869	4.12194	Guanine nucleotide-binding protein-like 3
KNOP1	-0.29865	3.12799	Lysine-rich nucleolar protein 1
STAT1	-0.297993	3.40809	Signal transducer and activator of transcription 1-alpha/beta
ZC3HAV1	-0.297541	3.71163	Zinc finger CCCH-type antiviral protein 1
RPS5	-0.296771	3.57127	40S ribosomal protein S5
NSUN2	-0.294598	5.87173	tRNA (cytosine(34)-C(5))-methyltransferase
WDHD1	-0.291812	4.0346	WD repeat and HMG-box DNA-binding protein 1
HECTD3	-0.291591	3.12093	E3 ubiquitin-protein ligase HECTD3
NAT10	-0.286347	3.2948	N-acetyltransferase 10
LRPPRC	-0.285965	3.98724	Leucine-rich PPR motif-containing protein, mitochondrial
TOMM40	-0.282547	3.33684	Mitochondrial import receptor subunit TOM40 homolog
PUS1	-0.278393	4.38408	tRNA pseudouridine synthase;tRNA pseudouridine synthase A, mitochondrial
HID1	-0.27839	4.46571	Protein HID1
FXYD3	-0.277665	4.18172	FXYD domain-containing ion transport regulator 3
DNAJC2	-0.272764	3.25515	DnaJ homolog subfamily C member 2
NNT	-0.272755	4.08218	NAD(P) transhydrogenase, mitochondrial
FKBP5	-0.272502	3.23964	Peptidyl-prolyl cis-trans isomerase FKBP5
RPL10	-0.271149	3.1899	60S ribosomal protein L10
DHX36	-0.270095	3.42728	Probable ATP-dependent RNA helicase DHX36
LARP4	-0.269266	3.67211	La-related protein 4
TRAFD1	-0.269172	4.09034	TRAF-type zinc finger domain-containing protein 1
TMED10	-0.267046	3.64014	Transmembrane emp24 domain-containing protein 10
PIP4K2C	-0.263657	3.40651	Phosphatidylinositol 5-phosphate 4-kinase type-2 gamma
XRN2	-0.259788	3.77379	5-3 exoribonuclease 2
EEF2	-0.259564	5.2302	Elongation factor 2
PSMC1	-0.25826	3.84706	26S protease regulatory subunit 4
TFRC	-0.256019	3.74425	Transferrin receptor protein 1
MCM4	-0.255777	3.60714	DNA replication licensing factor MCM4
MCM5	-0.255512	3.4097	DNA replication licensing factor MCM5
SKIV2L2	-0.254259	3.32057	Superkiller viralicidic activity 2-like 2
ANKRD28	-0.254233	3.75409	Serine/threonine-protein phosphatase 6 regulatory ankyrin repeat subunit A
CHD4	-0.251997	3.98829	Chromodomain-helicase-DNA-binding protein 4
PRPF6	-0.245268	3.50159	Pre-mRNA-processing factor 6
ALYREF	-0.237579	4.46268	THO complex subunit 4

ATP1B1	-0.233679	3.56518	Sodium/potassium-transporting ATPase subunit beta-1
HNRPLL	-0.23316	4.35933	Heterogeneous nuclear ribonucleoprotein L-like
STX12	-0.226731	4.04171	Syntaxin-12
RAB14	-0.223739	4.14955	Ras-related protein Rab-14
FLOT2	-0.216904	3.7788	Flotillin-2
Sep-09	-0.216505	4.38494	Septin-9
PSMC5	-0.211609	3.53747	26S protease regulatory subunit 8
PSMC2	-0.207065	5.25376	26S protease regulatory subunit 7
EIF4A1	-0.203064	5.02053	Eukaryotic initiation factor 4A-I
PFDN4	-0.200634	4.37441	Prefoldin subunit 4
PAPSS1	-0.19461	3.84453	Bifunctional 3-phosphoadenosine 5-phosphosulfate synthase 1
OGDH	-0.187538	4.06395	2-oxoglutarate dehydrogenase, mitochondrial
EIF4G1	-0.182456	5.95596	Eukaryotic translation initiation factor 4 gamma 1
CORO7	-0.174668	3.97001	Coronin-7;Coronin
PUF60	-0.172395	4.61392	Poly(U)-binding-splicing factor PUF60
MAT2A	-0.155081	4.31454	S-adenosylmethionine synthase isoform type-2
KIF5B	-0.154886	5.71176	Kinesin-1 heavy chain

Table S8. Significantly up-regulated proteins in DMOG

Gene names	log[ratio(H/L)	-log[*p* value]	Protein names
NDRG1	3.83531	7.24442	Protein NDRG1
BNIP3L	2.64923	5.03637	BCL2/adenovirus E1B 19 kDa protein-interacting protein 3-like
P4HA2	1.51736	5.55303	Prolyl 4-hydroxylase subunit alpha-2
HK2	1.39205	5.13745	Hexokinase-2
NCBP2	1.19983	6.41893	Nuclear cap-binding protein subunit 2
PLOD2	1.18135	5.57685	Procollagen-lysine,2-oxoglutarate 5-dioxygenase 2
P4HA1	1.04425	5.6735	Prolyl 4-hydroxylase subunit alpha-1
ERO1L	0.991414	4.83297	ERO1-like protein alpha
AK4	0.929949	3.79637	GTP:AMP phosphotransferase AK4, mitochondrial
ANKZF1	0.909652	4.51148	Ankyrin repeat and zinc finger domain-containing protein 1
LDHA	0.791535	6.04436	L-lactate dehydrogenase A chain
PGK1	0.742316	7.61788	Phosphoglycerate kinase 1;Phosphoglycerate kinase
GBE1	0.73468	6.25427	1,4-alpha-glucan-branching enzyme
POLR2H	0.619466	3.79452	DNA-directed RNA polymerases I, II, and III subunit RPABC3
IGF1R	0.595453	3.63693	Insulin-like growth factor 1 receptor
PPP2R2A	0.544925	4.23203	Serine/threonine-protein phosphatase
GYS1	0.54217	4.6619	Glycogen [starch] synthase, muscle
ALDOC	0.501259	5.46391	Fructose-bisphosphate aldolase C
TPI1	0.456526	4.54037	Triosephosphate isomerase
NOL3	0.449872	4.04272	Nucleolar protein 3
PFKP	0.446294	5.69721	6-phosphofructokinase type C
DSP	0.430348	5.52857	Desmoplakin
ALDOA	0.415263	4.78178	Fructose-bisphosphate aldolase A
TAGLN2	0.405527	3.4434	Transgelin-2
PNPO	0.405424	3.56248	Pyridoxine-5-phosphate oxidase
CPOX	0.367166	3.82201	Coproporphyrinogen-III oxidase, mitochondrial
ELMSAN1	0.364272	4.18548	ELM2 and SANT domain-containing protein 1
EIF4H	0.360228	3.82612	Eukaryotic translation initiation factor 4H
PKM	0.325008	6.45681	Pyruvate kinase isozymes M1/M2
PGAM1	0.310005	4.56633	Phosphoglycerate mutase 1
ANP32A	0.294952	3.46909	Acidic leucine-rich nuclear phosphoprotein 32 family member A
DARS	0.277904	5.29059	Aspartate--tRNA ligase, cytoplasmic
GSN	0.277655	4.82769	Gelsolin
OTUB1	0.267421	3.76272	Ubiquitin thioesterase OTUB1
HEBP2	0.259046	4.84815	Heme-binding protein 2
GAPDH	0.258315	6.88194	Glyceraldehyde-3-phosphate dehydrogenase
ENO1	0.254389	3.65464	Alpha-enolase
FAM129B	0.24519	3.74561	Niban-like protein 1
PFKL	0.194407	4.34217	6-phosphofructokinase, liver type
PSAP	0.16585	4.02795	Proactivator polypeptide;Saposin-A

Table S9. Significantly down-regulated proteins in DMOG

Gene names	$\log_2$[ratio((H/L)]	-log[*p* value]	Protein names
MLPH	-0.657918	4.98916	Melanophilin
KIAA0101	-0.625768	3.26198	PCNA-associated factor
FAM102A	-0.556088	3.86117	Protein FAM102A
PTMS	-0.524708	3.935	Parathymosin
SQSTM1	-0.520046	5.46136	Sequestosome-1
SDC1	-0.510042	4.73359	Syndecan-1
EEF1D	-0.479853	4.64609	
PRKAR1A	-0.478244	3.78297	cAMP-dependent protein kinase type I-alpha regulatory subunit;
HMGCS1	-0.464222	3.23901	Hydroxymethylglutaryl-CoA synthase, cytoplasmic

KPNA2	-0.455265	5.03837	Importin subunit alpha-2
TPRN	-0.411272	3.34876	Taperin
CSDE1	-0.396696	3.45475	Cold shock domain-containing protein E1
RPL36A	-0.390458	3.40076	60S ribosomal protein L36a
DDX5	-0.388337	4.95503	Probable ATP-dependent RNA helicase DDX5
CAPRIN1	-0.386978	5.12565	Caprin-1
EHMT1	-0.374902	4.66773	Histone-lysine N-methyltransferase EHMT1
BAZ1A	-0.360272	3.78302	Bromodomain adjacent to zinc finger domain protein 1A
RPS25	-0.35945	3.84239	40S ribosomal protein S25
RPL13	-0.355984	3.63935	60S ribosomal protein L13
PSMD6	-0.353648	3.47417	26S proteasome non-ATPase regulatory subunit 6
EIF3C	-0.319105	3.56467	Eukaryotic translation initiation factor 3 subunit C
DNAJA1	-0.311529	3.44158	DnaJ homolog subfamily A member 1
NACA	-0.306446	3.95203	Nascent polypeptide-associated complex subunit alpha
RPS19	-0.295525	3.72607	40S ribosomal protein S19
MAT2A	-0.29054	4.0044	S-adenosylmethionine synthase
EIF3K	-0.283197	3.61778	Eukaryotic translation initiation factor 3 subunit K
RANGAP1	-0.281118	4.48919	Ran GTPase-activating protein 1
EIF3H	-0.274564	3.79997	Eukaryotic translation initiation factor 3 subunit H
HSPH1	-0.271052	4.33491	Heat shock protein 105 kDa
SLC1A5	-0.260895	4.21106	Neutral amino acid transporter B(0)
PSMC2	-0.2482	6.79471	26S protease regulatory subunit 7
TGM2	-0.243489	3.62262	Protein-glutamine gamma-glutamyltransferase 2
YBX3	-0.23176	4.0282	Y-box-binding protein 3
CCT5	-0.229459	4.3471	T-complex protein 1 subunit epsilon
RPS4X	-0.223501	4.33435	40S ribosomal protein S4, X isoform
YBX1	-0.223048	4.62178	Nuclease-sensitive element-binding protein 1
EIF4A1	-0.215818	4.04397	Eukaryotic initiation factor 4A-I
RPL27A	-0.212129	4.38918	60S ribosomal protein L27a
HSPA8	-0.178096	4.2841	Heat shock cognate 71 kDa protein

Table S10. Significantly up-regulated proteins in DMOG relative to hypoxia (heavy/medium)

Gene names	log_2[ratio(H/M)]	-log[*p* value]	Protein names
NEDD4	1.09641	3.23273	E3 ubiquitin-protein ligase
SLC38A2	0.979866	4.22916	Sodium-coupled neutral amino acid transporter 2
NCBP2	0.903039	3.90766	Nuclear cap-binding protein subunit 2
SYPL1	0.797537	4.42418	Synaptophysin-like protein 1
HIST2H2BE	0.772049	3.16648	Histone H2B type 2-E
NDUFA4	0.754868	4.27878	NADH dehydrogenase [ubiquinone] 1 alpha subcomplex subunit 4
H1FX	0.728701	3.53682	Histone H1x
RPL29	0.646838	4.63099	60S ribosomal protein L29
ASAP2	0.630353	3.33456	Arf-GAP with SH3 domain, ANK repeat and PH domain-containing protein 2
CHTOP	0.580613	3.33712	Chromatin target of PRMT1 protein
TFF1	0.578906	3.88616	Trefoil factor 1
KIAA1324	0.478327	3.28398	UPF0577 protein KIAA1324
ANLN	0.460777	3.49523	Actin-binding protein anillin
RPL37A	0.450363	4.34835	60S ribosomal protein L37a
PDIA5	0.419772	3.35827	Protein disulfide-isomerase A5
DHX37	0.404798	4.04313	Probable ATP-dependent RNA helicase DHX37
BZW1	0.388108	4.16682	Basic leucine zipper and W2 domain-containing protein 1
NDUFS5	0.361848	4.36249	NADH dehydrogenase [ubiquinone] iron-sulfur protein 5
PRPF3	0.34766	3.26282	U4/U6 small nuclear ribonucleoprotein Prp3
SPG20	0.344967	3.55475	Spartin
EPCAM	0.330648	3.92511	Epithelial cell adhesion molecule
RPL19	0.320097	3.2588	Ribosomal protein L19;60S ribosomal protein L19
STXBP3	0.30705	3.87081	Syntaxin-binding protein 3
DDX5	0.301004	4.53656	Probable ATP-dependent RNA helicase DDX5
NELFCD	0.299833	3.41395	Negative elongation factor C/D
DLD	0.297744	4.68913	Dihydrolipoyl dehydrogenase, mitochondrial
C1QBP	0.276083	4.22206	Complement component 1 Q subcomponent-binding protein, mitochondrial
NSUN2	0.273122	3.99805	tRNA (cytosine(34)-C(5))-methyltransferase
EHD4	0.27028	3.81196	EH domain-containing protein 4
PTPRK	0.256331	3.52937	Receptor-type tyrosine-protein phosphatase kappa
MCM5	0.242821	4.16166	DNA replication licensing factor MCM5
FKBP5	0.219766	3.78821	Peptidyl-prolyl cis-trans isomerase FKBP5
PPM1G	0.207615	4.74102	Protein phosphatase 1G
LRPPRC	0.19948	3.83364	Leucine-rich PPR motif-containing protein, mitochondrial
IDH3B	0.198436	4.5958	Isocitrate dehydrogenase [NAD] subunit beta, mitochondrial
DDX46	0.192007	3.93077	Probable ATP-dependent RNA helicase DDX46

Table S11. Significantly down-regulated proteins in DMOG relative to hypoxia (heavy/medium)

Gene names	log_2[ratio(H/M)]	-log[*p* value]	Protein names
UBE2L3	-0.963726	3.22485	Ubiquitin-conjugating enzyme E2 L3
SNRPC	-0.851842	5.10765	U1 small nuclear ribonucleoprotein C
PTP4A2	-0.817034	3.27497	Protein tyrosine phosphatase type IVA 2
EIF3G	-0.70636	3.61992	Eukaryotic translation initiation factor 3 subunit G
PCMT1	-0.67357	3.16444	Protein-L-isoaspartate(D-aspartate) O-methyltransferase
SCP2	-0.659361	3.12181	Non-specific lipid-transfer protein
RPS21	-0.651334	3.07074	40S ribosomal protein S21
ZMYND8	-0.64398	4.27387	Protein kinase C-binding protein 1
CLIC3	-0.640185	4.36952	Chloride intracellular channel protein 3
NDUFA2	-0.632818	4.81218	NADH dehydrogenase [ubiquinone] 1 alpha subcomplex subunit 2
GIMAP2	-0.629313	3.65655	GTPase IMAP family member 2
ACYP1	-0.627096	3.58587	Acylphosphatase-1;Acylphosphatase
CHCHD2	-0.606751	4.13052	Coiled-coil-helix-coiled-coil-helix domain-containing protein 2
HSPE1	-0.593456	3.40459	10 kDa heat shock protein, mitochondrial
HNRNPH3	-0.591214	4.28594	Heterogeneous nuclear ribonucleoprotein H3
PFN1	-0.588556	4.97873	Profilin-1
PRDX5	-0.528434	5.74432	Peroxiredoxin-5, mitochondrial
TPI1	-0.518224	4.22579	Triosephosphate isomerase
SNAPIN	-0.504186	3.76315	SNARE-associated protein Snapin
CNN2	-0.502158	5.16771	Calponin-2
PEBP1	-0.486988	4.81447	Phosphatidylethanolamine-binding protein 1
GATAD2A	-0.475996	4.3728	Transcriptional repressor p66-alpha
EIF4H	-0.474162	3.83175	Eukaryotic translation initiation factor 4H
PPIA	-0.472656	4.25751	Peptidyl-prolyl cis-trans isomerase A;Peptidyl-prolyl cis-trans isomerase
SULF2	-0.472085	3.40119	Extracellular sulfatase Sulf-2
ARPC5L	-0.469343	3.11714	Actin-related protein 2/3 complex subunit 5-like protein
MSI2	-0.465592	3.25881	RNA-binding protein Musashi homolog 2
LGALS3	-0.457352	3.18792	Galectin-3
CWC15	-0.455812	3.39495	Spliceosome-associated protein CWC15 homolog
NOSIP	-0.450373	3.64726	Nitric oxide synthase-interacting protein

HNRNPH1	-0.449474	3.74767	Heterogeneous nuclear ribonucleoprotein H
COA6	-0.446661	3.83764	Cytochrome c oxidase assembly factor 6 homolog
AK2	-0.42834	3.23634	Adenylate kinase 2, mitochondrial
NOL3	-0.426934	4.28919	Nucleolar protein 3
PARK7	-0.418468	3.57327	Protein DJ-1
RBM4	-0.407416	4.06885	RNA-binding protein 4
SRP9	-0.386292	3.42568	Signal recognition particle 9 kDa protein
CCS	-0.383297	3.46201	Copper chaperone for superoxide dismutase;Superoxide dismutase [Cu-Zn]
BLVRB	-0.378092	3.53928	Flavin reductase (NADPH)
LGALS1	-0.376803	3.26032	Galectin-1
AKT1S1	-0.366907	3.34716	Proline-rich AKT1 substrate 1
Q14980-2	-0.34914	4.99016	
TGM2	-0.346451	4.01642	Protein-glutamine gamma-glutamyltransferase 2
DAZAP1	-0.345576	3.26674	DAZ-associated protein 1
TAGLN2	-0.341282	3.71901	Transgelin-2
ENO1	-0.319637	4.57198	Alpha-enolase
VASP	-0.310112	3.80508	Vasodilator-stimulated phosphoprotein
HEXIM1	-0.300394	3.57786	Protein HEXIM1
HSPB1	-0.299896	4.13041	Heat shock protein beta-1
ENO2	-0.283074	3.53315	Gamma-enolase;Enolase
HIBADH	-0.278648	3.57688	3-hydroxyisobutyrate dehydrogenase, mitochondrial
GRPEL1	-0.270308	4.34536	GrpE protein homolog 1, mitochondrial
CFL1	-0.269765	3.61482	Cofilin-1
C19orf21	-0.264334	4.0242	Uncharacterized protein C19orf21
PTMS	-0.258772	4.85988	Parathymosin
PFN2	-0.24546	3.5997	Profilin;Profilin-2
PPP1R9B	-0.232767	3.83141	Neurabin-2
RRM2	-0.223847	4.08206	Ribonucleoside-diphosphate reductase subunit M2
DPYSL2	-0.208676	3.57016	Dihydropyrimidinase-related protein 2
ELAVL1	-0.191836	3.61441	ELAV-like protein 1
EVL	-0.187401	3.64367	Ena/VASP-like protein
STAU1	-0.185617	4.24804	Double-stranded RNA-binding protein Staufen homolog 1
STRAP	-0.182738	3.69387	Serine-threonine kinase receptor-associated protein
FLYWCH2	-0.180193	3.88937	FLYWCH family member 2
CREB1	-0.169742	4.60422	Cyclic AMP-responsive element-binding protein 1
UBAP2L	-0.145686	4.16374	Ubiquitin-associated protein 2-like
PPP1R13L	-0.140679	5.40131	RelA-associated inhibitor
